# Extraction Strategies for Profiling the Molecular Composition of Particulate Organic Matter on Glacier Surfaces

**DOI:** 10.1021/acs.est.4c10088

**Published:** 2025-02-27

**Authors:** Runa Antony, Pamela E. Rossel, Helen K. Feord, Thorsten Dittmar, Martyn Tranter, Alexandre Magno Anesio, Liane G. Benning

**Affiliations:** † GFZ Helmholtz Centre for Geosciences, D-14473 Potsdam, Germany; ‡ National Centre for Polar and Ocean Research, Ministry of Earth Sciences, 403804 Vasco Da Gama, Goa, India; § University of Oldenburg, Institute for Chemistry and Biology of the Marine Environment, D-26046 Oldenburg, Germany; ∥ Department of Environmental Science, Aarhus University, 4000 Roskilde, Denmark; ⊥ Department of Earth Sciences, Freie Universität Berlin, 12249 Berlin, Germany

**Keywords:** supraglacial carbon dynamics, molecular profiling of organics, pigmented microalgae, mineral–organic matrix, ultrahigh-resolution mass spectrometry, snow and ice surfaces, Iceland, Greenland

## Abstract

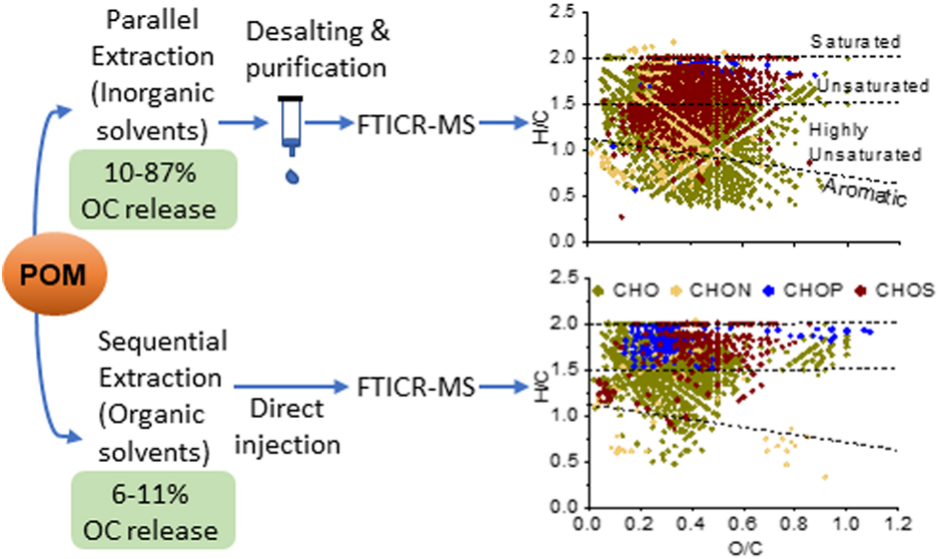

Pigmented microalgae thrive on supraglacial surfaces, producing “sticky” extracellular polymeric substances that combine into a mineral–organic matrix. Together, they enhance snow and ice melting by lowering the albedo. Understanding the chemical nature of particulate organic matter (POM) in this matrix is crucial in assessing its role in supraglacial carbon dynamics. We evaluated POM complexity in alga-rich snow and ice samples containing 0.3–6.4 wt % organic carbon (OC) via extractions with solvents of varying polarity, pH, and OM selectivity. Extraction yields were evaluated by OC analysis of the extracts, and the composition of extracted OM was analyzed using ultrahigh-resolution mass spectrometry. Individual hot water (HW), hydrochloric acid (HCl), and sodium hydroxide (NaOH) extractions achieved up to 87% efficiency, outperforming sequential, organic solvent-based extractions (<11%). OM extracted by HW, HCl, and NaOH combined had more molecular formulas (2827) than OM extracted with organic solvents (1926 formulas). Combined HW, NaOH, and HCl extractions yielded an OM composition with unsaturated, highly unsaturated, aromatic, and N-containing compounds, while unsaturated aliphatics and black carbon-derived polycyclic aromatics were enriched in the organic solvent extracts. This molecular profiling provides the first comprehensive insights into supraglacial POM composition, opening the window for understanding its role in the cryospheric carbon cycle.

## Abstract 2

This study details the crucial steps to characterize POM in alga-rich supraglacial snow and bare-ice habitats. It offers valuable insights into its compositions and influence on ecosystem dynamics in glacier environments.

## Introduction

Particulate organic carbon (POC) represents the largest reservoir of OC stored within glacier ice, with global estimates of the glacier POC store reaching approximately 1.39 PgC, and concentrations exceeding that of dissolved organic carbon (DOC) by an order of magnitude.^1,2^ In terrestrial cryospheric environments, diverse microbial communities, including pigmented microalgae, produce “sticky” extracellular polymeric substances (EPS) that bind cellular materials, organic matter (OM), and mineral dust into a mineral–organic matrix.^3,4^ In many other environments (e.g., soils, permafrost, or marine sediments),^5,6^ dissolved metals, salts, and nutrients are often bound to such organic-mineral matrix resulting in a complex particulate mixture. OM can attach to mineral surfaces through diverse mechanisms, encompassing sorption, complexation, ligand exchange, and chelation.^5,6^ The solubility of such particulate-bound OM (hereafter referred to as POM) in supraglacial aqueous settings, and its lability determine how much and what type of organic compounds are available for use by the microbial communities locally,^7^ and/or how much is transported to downstream ecosystems. Additionally, light-absorbing components of the POM, such as pigmented algae, black carbon, and minerals, are responsible for darkening snow and ice surfaces, significantly reducing surface albedo and accelerating melting.^8−14^ However, we lack information, on the chemical composition and reactivity of this POM. This information is crucial for (1) unraveling its capacity to support heterotrophic microbial communities, (2) assessing carbon dynamics on glaciers and ice sheets, and (3) understanding their contribution to ice melt. Moreover, such information is highly relevant and urgently needed due to the expansion of “dark zones” on glacier and ice sheet surfaces. This darkening accelerates meltwater fluxes,^9,14,15^ which are anticipated to increase further in future climate warming scenarios. Glacier and ice sheet surfaces are hydrologically connected to various surface, englacial, and subglacial environments^16,17^ that have distinct redox, ionic strength, and pH conditions.^18,19^ The strength of the mineral–organic interactions is sensitive to these conditions,^6,20^ and hence POM transport along such hydrological flow paths will impact OC accessibility for heterotrophic microorganisms and photochemical activity. The transformation of OM in supraglacial freshwater systems will also regulate the quantity and quality of OM delivered to the ocean by glacier runoff,^21^ which is projected to release two times more POC than DOC to downstream ecosystems in the future.^1^ POM on ice surfaces has been identified as a potential source of biolabile dissolved OM (DOM).^22^ Furthermore, approximately 10% of the total POM flux is considered labile,^7,23^ with the capacity to impact microbial community and activity.^24,25^ Yet, we lack fundamental knowledge of the chemical nature, and the factors controlling the stability and dynamics of POM in supraglacial environments. POM in soils and sediments represents a dynamic fraction of organic matter, closely associated with mineral components and influencing processes such as carbon sequestration and nutrient cycling. To study this OM, various chemical methods have been employed.^26−31^ These include the use of multiple solvents with different polarities^28,30,31^ or of solvents with varying pH to effectively separate OM from mineral components.^27,28^ Organo-mineral interactions are crucial for OM stability^27^ and extractions that effectively separate OM from minerals and other components are essential. This is particularly important on glacier surfaces, where EPS-producing microbes bind minerals, dust, and soot within an organic matrix,^3,4^ which contributes to the POM pool. However, a method is lacking to extract and characterize biomass-rich snow and ice particulate samples, significantly limiting our molecular-scale understanding of POM in these habitats. To fill this knowledge gap, we systematically tested and validated sequential extraction methods using organic solvents of various polarities and parallel extractions using inorganic solvents of varying pH. Our aim was to quantitively evaluate the most efficient method for recovering and characterizing OM in particulate-rich snow and ice samples and to disentangle the molecular intricacies of the particulate-bound OC pools.

## Materials and Methods

### Sample Description

We characterized POM from natural supraglacial, alga-rich “red snow” or “dark ice” samples from Iceland^32,33^ and Southeast Greenland,^34,35^ and from an artificial mineral–organic reference material. The reference material contained an analog mineral mix that mimics the mineral composition of Icelandic glaciers^36^ and the Greenland ice sheet^37^ combined with OM from EPS-producing microalgae cultures and a reference graphitic carbon (USGS24). For collection, processing, and details of the natural samples, and the information about the mineral–organic reference material, see Table S1, Supporting Information.

### Sequential Extractions

**Figure 1:**
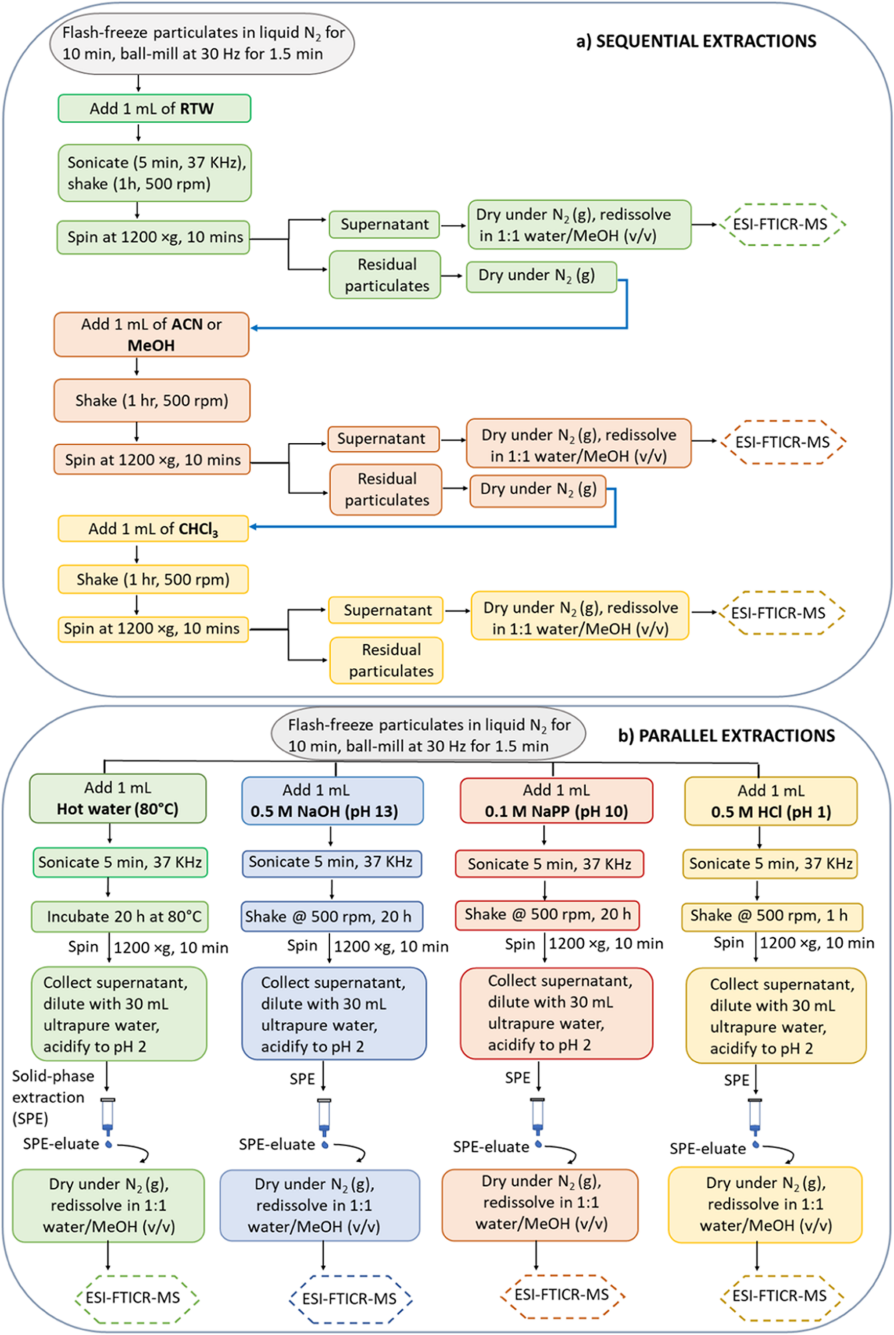
Experimental scheme for extracting OM from particulates by (a) sequential extraction steps using room temperature water (RTW)- acetonitrile (ACN)-chloroform (CHCl_3_), or RTW-methanol (MeOH)–CHCl_3_, and (b) parallel extraction with hot water (80°C, HW), sodium hydroxide (0.5 M NaOH), sodium pyrophosphate (0.1 M NaPP), and hydrochloric acid (0.5 M HCl). Extracted compounds are analyzed using electrospray ionization Fourier transform ion cyclotron resonance mass spectrometry (ESI-FTICR-MS). Residual particulates refer to the solid material remaining in the vial after centrifugation and removal of the supernatant which contains the extracted components.

We tested two sequential extraction protocols (modified after Tfaily et al.^31^) with 3 × 100 mg of milled particulates (and 3 × blanks): (1) room temperature water (RTW)- acetonitrile(ACN)-chloroform(CHCl_3_) and (2) RTW-methanol(MeOH)–CHCl_3_ (Figure 1a, Supporting Information). In both sequential extraction protocols, we progressed from polar to nonpolar solvents, as this solvent order has been found to provide the highest number and diversity of organic compounds extracted from soils and sediments.^31^ We refer to the fraction of organic carbon solubilized into the extraction solution as extractable DOC (DOC_ex_) throughout the manuscript. The extracts were dried and redissolved in a 1:1 mix of ultrapure water/MeOH (v/v) to yield a DOC_ex_ concentration of 5 mg C L^–1^. These samples were analyzed on a 15T Fourier transform ion cyclotron resonance mass spectrometer (FTICR-MS), coupled with electrospray ionization (ESI) by direct injection^31^ (see Supporting Information). Direct injection into the mass spectrometer was preferred over purification via solid-phase extraction (SPE), because of the low concentration of inorganic salts in the organic solvent extracts.^31^ The extraction efficiency was quantitatively estimated (see Supporting Information) from the initial total organic carbon (TOC) wt % of the particulates (Thermo, EA Isolink elemental analyzer) and the DOC_ex_ concentrations (Shimadzu TOC-L_CSH_ analyzer). Full details on the sequential extraction protocol and solvent selectivities are provided in the Supporting Information under the section “Sequential extractions”.

### Parallel Inorganic Solvent Extractions

We used four solvents with varying pH and selectivity (Table S2) for parallel extractions: (1) hot water (HW, 80°C; pH 6), (2) hydrochloric acid (HCl, 0.5 M; pH 1), (3) sodium pyrophosphate (NaPP, 0.1 M, pH 10), and (4) sodium hydroxide (NaOH, 0.5 M; pH 13). These solvents were selected as they extract distinct OM fractions (e.g., water-soluble, mineral-associated, OM with acidic functional groups) by targeting specific OM sorption mechanisms (Table S2, Supporting Information text: “Parallel extractions”). Each extraction was carried out in triplicate with 100 mg of milled particulates and blanks (Figure 1b). The OM extracted using inorganic solvents have high salt and metal concentrations,^26,27,38,39^ and therefore, we desalted and purified the extracts using SPE (100 mg, Agilent Bond Elut PPL cartridges).^40^ The SPE eluate was evaporated to dryness and reconstituted in a 1:1 mixture of ultrapure water and MeOH (v/v) to achieve a DOC_ex_ concentration of 5 mg C L^–1^, prior to FTICR-MS analysis.

### ESI-FTICR-MS Analysis and Data Processing

Molecular characterization was carried out using a Solarix 15T FTICR-MS (Bruker Daltonic) equipped with an Apollo II (Bruker) electrospray ionization source operating in negative ion mode. A total of 237 mass spectra were generated that were externally and internally calibrated using the arginine cluster and known molecular mass peaks in the sample over the entire mass range from 100 to 1000 *m*/*z*. Following calibration, molecular formulas above the method detection limit (MDL) of 3 were assigned using ICBM-OCEAN.^41^ Molecular formulas were assigned with an error of <0.5 ppm for the following combinations of elements: C_0–100_, O_0–50_, H_0–200_, N_0–4_, S_0–2_, and P_0–1_.

Molecular formulas were filtered to exclude those unlikely by applying the N, S, P rule and other filtration criteria such as disallowing the combination of >3 N, S, or P atoms per molecule, and formulas with elemental ratio O/C = 0 and O/C > 1.1; and H/C > 2 and N = 0.^41,42^ The homologous series network approach was applied to remove double assignments, considering CH_2_, CO_2_, H_2_, H_2_O, and O.^43^ Only formulas that were present in all four replicate measurements of each sample were considered, and their intensity was averaged for further evaluation. To remove potential contaminants, formulas in the method blanks (even if present in only one replicate) were excluded from the sample list. We calculated the modified aromaticity index (AI_mod_) and double-bond equivalents (DBE)^44,45^ and classified the identified molecular formulas into four broad chemical compound classes:^41^ saturated (DBE = 0), unsaturated (1.5 ≤ H/C ≤ 2), highly unsaturated (H/C < 1.5, AI_mod_ < 0.5), and aromatics (AI_mod_ > 0.5). Identified molecular formulas were assigned to polycyclic aromatics consistent with combustion-derived black carbon if AI_mod_ ≥ 0.67 and C ≥ 15.^46^ We also used the molecular lability boundary (MLB) approach^47^ to classify formulas into labile (MLB_L_, H/C ≥ 1.5) and recalcitrant (MLB_R_, H/C < 1.5) fractions and calculated peak intensity-weighted averages of elements (N_w_, S_w_, P_w_), and other parameters (H/C_w_, O/C_w_, DBE_w_, *m*/*z*_w_, AI_mod,w_). Full details for FTICR-MS analyses and data processing are given in the Supporting Information.

## Results and Discussion

### Extraction Efficiencies

The sequential extraction of the natural samples with RTW-ACN–CHCl_3_ produced the highest DOC_ex_ concentrations in the RTW extraction, followed by ACN and CHCl_3_, with extraction efficiencies between 8 ± 1 and 11 ± 0.2% (Table S3, Figure S1a). Sequential extraction with RTW-MeOH–CHCl_3_ recovered between 6 ± 0.1 and 10 ± 1% of the TOC present in the particulates (Table S3). Of the total DOC_ex_ recovered, the majority was extracted using RTW (76–90%), followed by MeOH (10–24%), with negligible contributions from the CHCl_3_ step (Table S3, Figure S1b). Similar trends were obtained using the reference mineral–organic mixture. The RTW-ACN–CHCl_3_ and RTW-MeOH–CHCl_3_ extraction efficiencies were slightly higher, but of the same order of magnitude as in other studies using similar solvents and extraction procedures.^30,31^ A higher extraction efficiency with RTW may imply a predominance of water-soluble or polar organic substances in our particulates. Studies of soil-bound OM revealed that ∼25% of the OM, mainly comprising aliphatics, carbohydrates, and carbonyl carbons at the soil-water interface, are readily mobilized in water.^48^ In contrast, the less polar organic solvents have a higher affinity for hydrophobic organic compounds of similar polarity that are more loosely bound to mineral surfaces^30,31^ through weak physical forces, such as van der Waals forces, or are held on the surface without strong bonding.^5,48^ The low extractability with these organic solvents suggests that this fraction of the OM in our samples is tightly bound to mineral surfaces through stronger interactions, potentially involving ligand exchange, electrostatic binding, cationic bridging, or covalent bonds^5,48^ and thus, more difficult to remove from the mineral matrix.^48,49^

The parallel extractions (Figure 1b) yielded up to 8 times greater OC concentration (i.e., OC yield) than those obtained using the sequential extractions (between 10 ± 1 and 87 ± 4% of the TOC; Table S4). In particular, the alkaline NaOH extractions recovered the highest amount of OC from all sample types (Figure S2). Extraction efficiency generally followed the order NaOH > HW > HCl > NaPP, except for the mineral–organic mix, which exhibited a slightly higher OC yield with HW than NaOH (Figure S2). The high OC yield from the alkaline NaOH (pH 13) extractions (39–87%) implies the presence of abundant acidic functional groups prone to deprotonation at higher pH. This leads to the electrostatic repulsion of OM from negatively charged soil minerals, thereby disrupting the strong bonds that bind OM to mineral surfaces.^27,50^ This is particularly pertinent to our particulates, sourced from alga-rich snow and ice habitats, where EPS exuded by algae significantly influences the binding of mineral particles. Carboxyl, amino, phosphate, sulfhydryl, phenolic, and hydroxyl functional groups in EPS exhibit pH-dependent protonation/deprotonation reactions.^51−53^ Environmental pH values higher than the p*K*_a_ value of the functional groups significantly change their charge, thereby influencing the adhesion behavior and conformation of the EPS structure.^54,55^ The alkaline NaPP extractions (pH 10) extracted less OC (17–48%), possibly because sodium pyrophosphate acts as a strong chelating agent, releasing OM bound to Ca^2+^ and metal ions, such as Fe^3+^ and Al^3+^.^26,27^ These results suggest that some of the OM likely formed metal-OM complexes bound to minerals. However, it is important to consider that the complexity of the mineral–organic matrix and the variety of possible interactions mean that our extractions involve a wide range of overlapping processes and reactions affecting the separation of OM from mineral components.

Interestingly, HW extracted between 12 and 68% of the TOC (Table S4). Previous studies have demonstrated the high extractability of organic carbon in HW compared to that at room temperature for soils^56−58^ and marine sediments,^59^ an observation that our data also confirm. Temperature-induced increases in OM released from the particulates are primarily driven by increased silicate mineral solubilities.^39^ At high temperatures, highly charged OM will desorb from mineral matrixes in exchange for OH^–^ to balance the solubilized cations.^39^ The HW-extractable OC fraction is strongly associated with labile organic compounds, characterized by fresher, less degraded constituents.^59,60^ This fraction is closely linked with DOM in the surrounding aqueous milieu, signifying a readily available form of carbon for microbial utilization and transport within diverse supraglacial environments during snow/ice melt.^61^ Similarly, the relatively high extractability of OC with HCl (10–68%; Table S4), especially from the particulates from Iceland, indicates that a large fraction of the OM bound to minerals is sensitive to acid hydrolysis and may be associated with poorly crystalline silica-, aluminum- or iron-bearing phases,^28,62^ as documented in the mineralogy of our samples.^4,36^ The extractions with the HW, HCl, and NaOH individually exhibited 2 to 15 times higher OC yields than the cumulative extraction efficiency of the sequential extraction methods. However, it is essential to note that a portion of the extracted OM was lost during the SPE desalting and purification (Figure 1b). This loss, while inherent to the process, was imperative to eliminate inorganic components that could potentially interfere with the FTICR-MS analysis.^63,40^ Specifically, 19 to 49% of the HCl-, 31 to 36% of the NaOH-, and 23 to 59% of the HW-extractable fractions were recovered in the SPE fraction.

When comparing OC extracted from our natural samples, our data shows up to three times more OC extraction efficiencies from Iceland than from the Greenland samples, regardless of the solvents used (Table S4). This difference in extractability between samples from both locations is likely due to the distinct mineralogical composition of the particulates. In Iceland, the particulates contain primarily basaltic volcanic ash,^36,64^ while the particulates from Greenland mainly consist of glacier flour made up of quartz, feldspars, clay, and hydroxyapatite.^4,37^ Differences in the mineral composition control the mechanisms and strength of the OM-mineral bonds.^5^ This, in turn, influences the stability^5^ and extractability of organic compounds.^65^ Also, the large specific surface areas, high reactivity, and solubility of poorly crystalline phases in volcanic ash and glass make these much more efficient and effective for sorption and desorption.^66^ This may explain why we observed a higher extractable OC fraction in the Iceland samples than the typical glacier flour-type material deposited on the Greenland ice sheet.^37^ These differences will invariably influence the OM binding and decomposition in these two locations. The effect of the interaction between the extraction solvents and the mineral–organic particulates is apparent in the case of the mineral–organic reference material, which, unlike the natural samples, exhibited a slightly higher OC yield with HW than NaOH (67 vs. 64%). This is a consequence of the differences in the OM binding mechanisms to the minerals in natural samples vs. the mineral–organic reference material. Our reference material cannot fully represent the natural mineral–organic matrix. It lacks the complexity of binding mechanisms inherent in a natural sample, where a succession of biotic and abiotic reactions under dynamic environmental conditions creates highly stabilized mineral–organic mixtures. This results in organic-mineral interactions and chemical diversities that are far more complex than that prepared in the laboratory, invariably, impacting the extractability of OM.

### Validation of the Extraction Protocols through Molecular Composition Analysis

We validated our method based on extraction efficiencies (determined quantitatively) and the characterization of OM in the artificial mineral–organic reference material. By analyzing the molecular compositions of the extracted OM using FTICR-MS (Table S5), we assessed whether it represented the components (algal biomass and pyrogenic carbon) in the reference material. We generated a composite list of molecular formulas for each extraction by combining the unique formulas from each solvent with those common between them. Among the formulas in the composite parallel HW, NaOH, and HCl extractions, 52% were labile (H/C ≥ 1.5),^47^ unsaturated aliphatic compounds that contained sulfur, nitrogen, and phosphorus, and 43% were highly unsaturated compounds (Table S5). The remaining formulas were aromatic (<5%) and polycyclic aromatic (0.5%) compounds, with more than 15 C atoms consistent with black carbon-derived OM. For both sequential extractions, the distribution of the compound categories was fairly similar, with unsaturated aliphatics, highly unsaturated and aromatic compounds accounting for 46–55, 32–36, and 13–17% of the molecular formulas, respectively. The proportion of polycyclic aromatics with more than 15 C atoms varied from 3 to 4% of the total identified formulas (Table S5). A large proportion (>80%) of the identified compound categories relate to algal biochemical compositions and metabolic processes. For example, highly unsaturated compounds have been linked to phytoplankton inputs in surface seawater DOM^67^ and are a major component of microalgae and their exudates,^68,69^ including cold-adapted microalgae.^70^ In particular, the thylakoid membrane systems in microalgal chloroplasts contain high percentages of highly unsaturated fatty acids.^71,72^ Their proportion increases upon blue-green light exposure and is coupled with increased photosynthetic pigment production,^73^ due to the rearrangement of the microalgal chloroplasts in response to light levels.^74,75^ The glacier ice algae that are dominant in our ice samples produce photoprotective pigments in response to UV and high-energy blue visible radiation, offering protection to cells.^10^ On bare-ice surfaces, these algae constitute a major light-absorbing component and are responsible for the darkening and up to 26% albedo decline associated with glacier ice algae blooms.^10,14^ Microalgae also produce many unsaturated aliphatic compounds, such as fatty acids, which form a component of membrane lipids, serving multiple biological roles such as nutrient storage and cell signaling.^76^ In our samples, 21% of the unsaturated aliphatics had O/C < 0.6 and H/C > 1.7 consistent with aliphatic molecules in microalgal biomass.^77−79^ Aromatic compounds, such as aromatic amino acids^80^ and aromatic pigments,^81^ are also integral components of algal biomass. Detection of these diverse compound classes signifies that our extractions released the major algal biomolecular components and metabolites. In addition, we detected polycyclic aromatics containing >16 C atoms (Table S5), with high DBE values (>13) and high AImod (>0.70), suggesting the presence of pyrogenic-derived OM.^46,82^ This indicates that despite the low solubility of graphitic OM,^83^ and generally poor ionization efficiency in ESI,^84^ our methods (particularly the sequential extraction) extracted a fraction of this potentially pyrogenic material (Table S5). Our results are corroborated by a recent study that detected polycyclic aromatics from artificially generated soot emission particulate matter extracts under standard ESI-FTICR-MS conditions and confirmed by laser desorption ionization (LDI) mass spectrometry.^82^ The latter method is particularly sensitive to detecting polyaromatic hydrocarbons on soot.^85^ Overall, the diverse compound classes detected from our artificial mineral–organic mixture affirm the effectiveness of the employed extraction methods in releasing organic constituents that represent the added algal biomass and pyrogenic carbon source, thus validating the method. Microalgal biomolecular components and metabolites detected in our natural samples suggest that our methods capture the inherent diversity of POM in these habitats (see section ‘POM composition of supraglacial snow and ice samples’). To our knowledge, our study represents the first attempt to establish optimal extraction conditions and validate a method for characterizing POM in snow and ice settings.

### Molecular Characteristics of OM Obtained Using Different Extraction Protocols

#### Sequential Extractions

**Figure 2:**
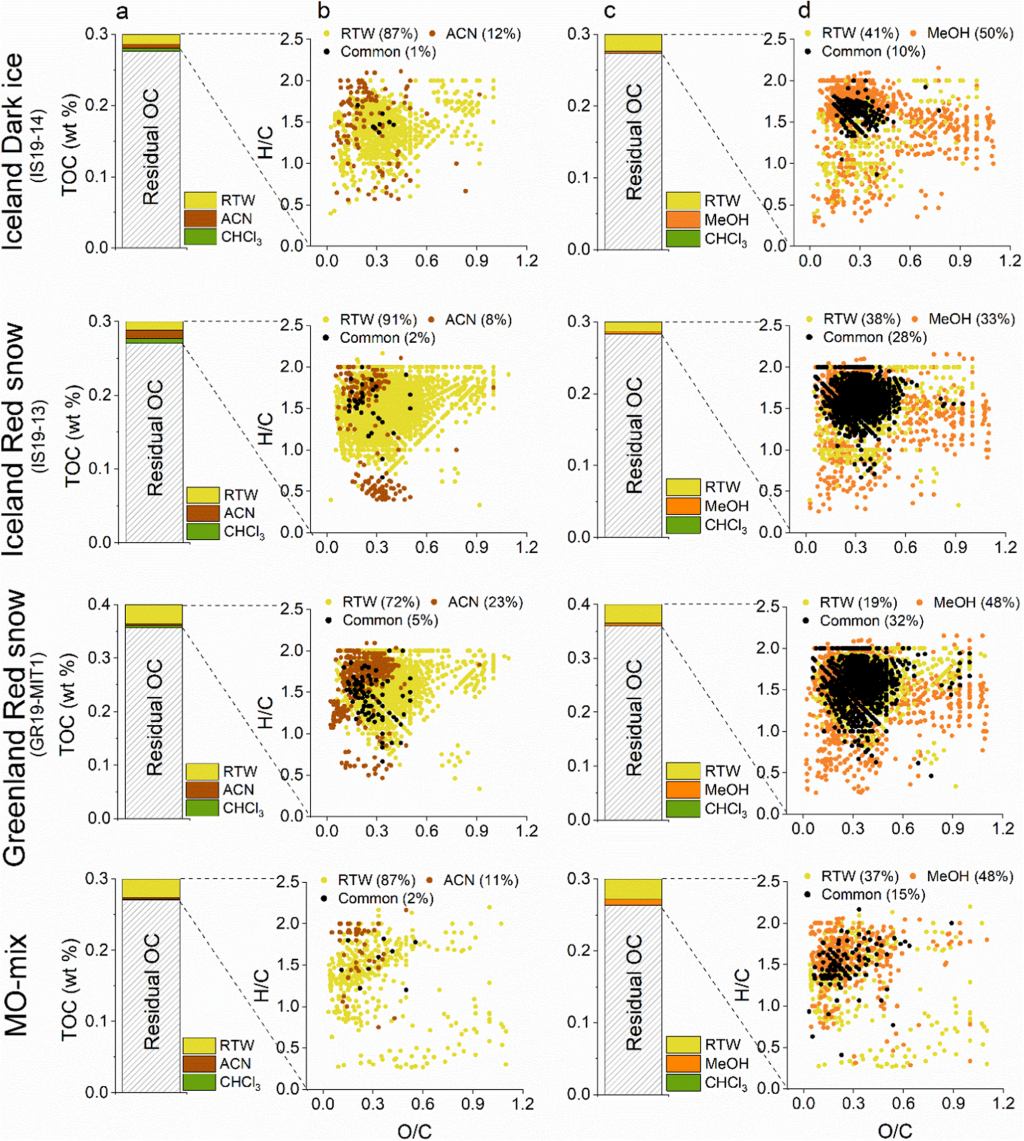
Comparison of the total organic carbon (TOC in wt %) in the particulates that were extracted using the sequential extractions with room temperature water (RTW)- acetonitrile (ACN)-chloroform (CHCl_3_), or RTW-methanol (MeOH)–CHCl_3_ versus the residual OC (columns a and c) for the samples from Iceland, Greenland, and the mineral–organic mix (MO-mix). The residual OC is the fraction of the TOC in the particulates that remains insoluble or inaccessible to the solvents under the applied extraction conditions. The corresponding van Krevelen plot of the molecular formulas unique to each extract and common between extracts are shown in columns b and d. Values in parentheses denote the percentage of molecular formulas that were unique or common between those identified in each extract. In the CHCl_3_ extracts, no ionizable peaks were detected in some extractions, while in others, the small number of ionizable peaks that could be assigned molecular formulas were excluded during the blank correction.

The sequential extractions using RTW-ACN–CHCl_3_ showed that the water extracts yielded a substantially higher number of molecular formulas (799 to 1953) than ACN (120 to 463), consistent with the up to 26 times more DOC_ex_ concentrations in the water extracts (Table S3, Figure S1a). Between 2 and 34 molecular formulas, predominantly oxygen-poor (O/C ≤ 0.5) unsaturated compounds, were detected in the CHCl_3_ extracts across all four replicate measurements for each sample. However, these formulas were excluded during the blank subtraction step. Additionally, no stable signal was obtained for the CHCl_3_ extracts in the RTW-MeOH–CHCl_3_ extraction, and therefore no mass spectrum could be acquired. Thus, we will refer to these extractions as RTW-ACN and RTW-MeOH, respectively. The RTW extracts in the RTW-ACN extraction exhibited the highest number of unique molecular formulas, with 72 to 91% specific to this extract (Figure 2b), and a greater molecular diversity reflected in the wide range of chemical classes represented. The RTW extracts were dominated by a high relative abundance of highly unsaturated compounds (32–52%) and primarily oxygen-poor unsaturated aliphatics (42–66%). Aromatics and polycyclic aromatics were one and 2 orders of magnitude lower in relative abundances, respectively, compared to highly unsaturated compounds and unsaturated aliphatics. Conversely, ACN was more selective for aromatic compounds, including polycyclic aromatics (Figure S3a), particularly in the red snow samples from Greenland and Iceland. ACN extracts were also more enriched in unsaturated aliphatics (53–71%) than the RTW extracts. The peak intensity weighted average mass of the molecular formulas identified in the RTW extracts was lower (*m*/*z*_w_ 331) than those in the ACN extracts (*m*/*z*_w_ 463) but RTW-extracted molecular formulas exhibited similar ratios of O/C_w_ and H/C_w_ as ACN (RTW: O/C_w_ 0.4, H/C_w_ 1.6; ACN: O/C_w_ 0.2, H/C_w_ 1.4). It is important to note that ionization efficiency, charge competition, and matrix effects can influence peak intensities when interpreting peak intensity-weighted data. This may lead to the suppression of compounds with lower abundances and ionization efficiencies.^86,87^ Compared to RTW extracts, the MeOH extract generally contained more unsaturated aliphatics (64–71%) and aromatic compounds (5–9%, Figure S3b). These findings align with those of Tfaily et al.,^31^ who demonstrated that the water fractions of soil and sediment OM contained the highest diversity of molecular formulas, and the ACN and MeOH fractions contained higher percent abundances of unsaturated compounds similar to lipids. MeOH extracted organic compounds with *m*/*z*_w_ 417, O/C_w_ 0.3, and H/C_w_ 1.6.

We combined the unique molecular formulas extracted by each solvent and the molecular formulas that overlapped between the solvents to generate a composite list of molecular formulas for each sequential extraction protocol, as a means of comparing the RTW-ACN and RTW-MeOH extractions (Tables S6 and S7). The molecular formulas detected in the RTW-ACN extraction protocol were marked by a 3 times higher peak intensity-weighted abundance of P-containing molecular formulas and up to 1.5 times higher degrees of unsaturation (DBE_w_) and aromaticity (AI_mod,w_) than those in the RTW-MeOH extraction (Table S6). Highly unsaturated compounds were abundant in the RTW-ACN extraction (Table S7), while polycyclic aromatics and oxygen-poor unsaturated aliphatics were generally more abundant in the RTW-MeOH extraction. Similar trends were observed with the mineral–organic mix, except that the black carbon-derived polycyclic aromatics were more enriched in the RTW-ACN extraction. The divergent compositions of OM bound to particulates following the sequential extractions can be explained by considering solvent selectivity and interactions with specific functional groups. Water being a polar solvent, can extract a wide range of polar organic molecules, while the nonpolar ACN preferentially extracts compounds of similar polarities, such as hydrophilic aromatic compounds.^30,31^ MeOH has a polarity between that of water and ACN and extracts both water-soluble and less polar OC pools, resulting in substantial compositional overlap between these pools.^30,31^ Overall, each solvent extracted a unique pool of compounds, with fewer peaks common between RTW and ACN than between RTW and MeOH (Figure 2). This underscores the importance of using multiple solvents in sequential extraction to capture the broadest spectrum of organic compounds bound to the particulates.

### Parallel Inorganic Extractions

The OC extraction efficiencies using the two alkaline solvents, NaOH and NaPP, revealed a consistently lower OC yield with NaPP than NaOH across all samples (Table S4), as determined by the amount of OC extracted relative to the TOC content of the particulates. A detailed comparison of the mass spectra for one sample underscored this trend, wherein NaOH extracted a 4-fold higher number of unique formulas than NaPP, with an approximate 41% overlap between the two solvents (Figure S4). Indeed, NaOH captures a substantial portion (79%) of the organic compounds that NaPP extracts. The remaining compounds in the NaPP extract, not captured by NaOH, were recovered in the HW and HCl extracts. Only 4% of the compounds in the NaPP extract, mainly CHO and CHOS, were not represented in any of the extracts (for molecular formulas in the NaOH and NaPP extracts see Table S8). The substantial overlap (Figure S4) and the superior extraction capabilities of NaOH (Table S4) allowed us to exclude NaPP from subsequent mass spectrometry analyses. Alkaline extractions, especially NaOH, have been used for decades to elucidate the chemical structure of OM in soils.^29,88^ Concerns have been raised regarding the potential for chemical alteration during alkali extraction,^89^ as the existence of alkali-extracted “humic substances” from soil has not been verified by direct *in situ* measurements.^90^ These concerns have been addressed in recent years where it has been shown that NaOH extractions cause very little modification of OM, thereby closely representing natural OM components in soil and water.^29,91^ Additionally, NaOH seems more effective at solubilizing our volcanic glass and silicate mineral fractions, especially poorly crystalline ones, thereby more readily releasing the organic compounds associated with the mineral particles.

**Figure 3:**
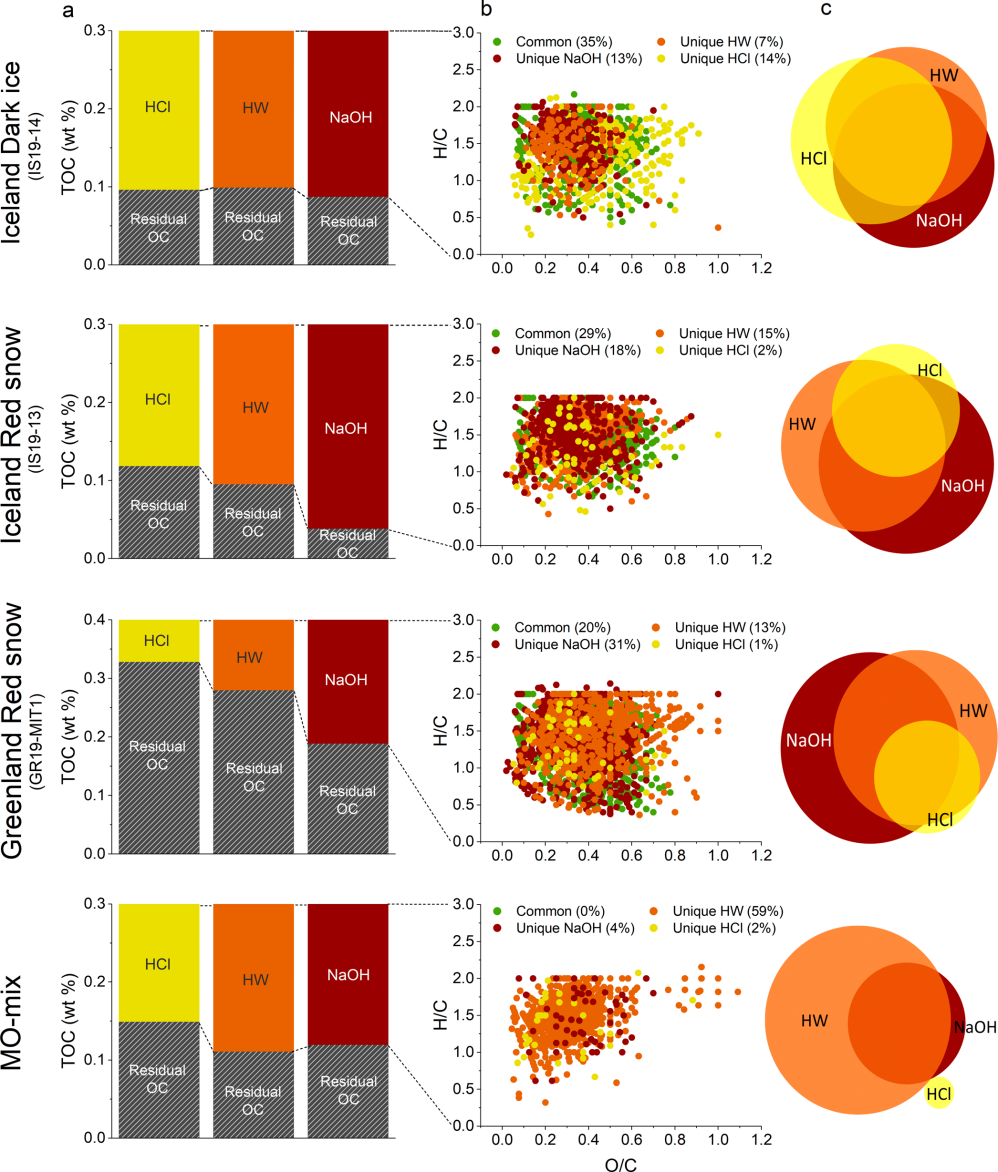
(a) Proportion of total organic carbon (TOC) extracted by each solvent versus the proportion unextracted (Residual OC) from the particulate matrix for the samples from Iceland, Greenland, and the mineral–organic mixture (MO-mix). (b) van Krevelen diagrams for OM unique to HW (orange), NaOH (brown), and HCl (yellow) extracts and common (green) between any two or all three extracts. (c) Scaled Venn diagram of the proportion of unique and common molecular formulas identified in the extracts. The area of each circle is proportional to the number of molecular formulas it contains. Areas of overlap indicate molecular formulas that appear in two (or all three) samples. Areas of no overlap indicate molecular formulas unique to that individual type of extract. Refer to Table S9 for details on the number of molecular formulas common between different solvents and Table S10 for the peak intensity weighted bulk properties and the number of identified molecular formulas unique to HW, NaOH, and HCl extracts.

The molecular formulas detected in the HW, HCl, and NaOH solvent extracts from all snow, ice, and mineral–organic mix samples showed an overlap of 20–35% (Figure 3, Table S9). It should be noted that identical formulas in the different extracts do not necessarily indicate identical molecular structures, as numerous isomers may exist per molecular formula.^92^ Each extraction yielded a distinctive molecular composition, resulting in a better representation of the chemical diversity inherent to the natural samples (Figure 3b,c). NaOH extracts exhibited a higher number of unique molecular formulas with 13 to 31% of molecular formulas (252 to 1254 formulas) being unique to this solvent, followed by HW (134–523 formulas, 7 to 15%), and HCl extraction (44–270 formulas, 1 to 14%). The mineral–organic mix exhibited a different trend (Figure 3c, bottom row), likely due to the higher complexity of OM binding in natural samples compared to those in the artificial reference material. In these parallel extractions, the effect of the extraction solvent differed based on sample type. Specifically, HW and NaOH extracted 3 times more unique formulas (HW: 387–523 and NaOH: 461–1254 formulas) from red snow than dark ice (Table S10). In contrast, HCl uniquely extracted 5 times more molecular formulas (270 formulas) from the dark ice sample than the red snow samples (44–61 formulas). Overall, distinctive compositional patterns unique to each solvent were observed. Notably, in the red snow samples, NaOH extracts showed a high relative abundance of highly unsaturated compounds (43–48%), but in the ice sample, the unsaturated aliphatics (60%) dominated. HCl uniquely extracted many aromatic compounds from all samples (Figure S5). Among the molecular formulas unique to HW extracts, unsaturated aliphatics (44–60%) and highly unsaturated compounds (32–45%) were predominant.

HW extracts also contained several N- (CHON, 15–29%) and S-containing compounds (CHOS, 11–37%) across all samples. P-containing compounds (CHOP, 2–8%) were detected exclusively in the red snow samples. The molecular formulas unique to NaOH extracts in all the samples were characterized by a higher average molecular mass (avg *m*/*z*_w_ 454) than those extracted by HCl, which exhibited an average *m*/*z*_w_ of 278. Molecular formulas detected in the HW extracts had an intermediate average molecular mass of *m*/*z*_w_ 326. All three solvents extracted organic compounds with an average molar ratio of H/C_w_ 1.5 and O/C_w_ 0.4. Using the lability index MLB_L_^47^ we could show that HW and NaOH extracted a more labile OM fraction (MLB_L_ of ∼53%), while HCL-extracted OM had an average MLB_L_ of 36%. The unique compound classes extracted by each solvent suggest that the particulates harbor a chemically diverse pool of OM. Different solvents target specific fractions of this diversity, revealing distinct chemical profiles highlighting the heterogeneity of the OM. This indicates that a multisolvent approach ensures a more thorough representation of the organic composition, as it accounts for multiple interactions and affinities of different solvents with various organic components within the samples.

### Integrated Evaluation of Sequential and Parallel Extraction Results

We evaluated the composite list of molecular formulas for the parallel extractions, generated by combining the unique molecular formulas extracted by HW, NaOH, and HCl and the molecular formulas that overlapped between these solvents (Table S11), with the composite list generated for the sequential extraction protocols (Figure S6, Table S7). While the data sets from the parallel and sequential extraction protocols may exhibit inherent biases due to differences in their targeted OM pools, extraction methodologies, and sample processing, these variations do not undermine the broader objective of this study. By evaluating these different data sets, together with extraction efficiencies, we aimed to determine effective strategies for maximizing OM recovery and providing a comprehensive characterization of the diverse organic fractions present in particulate-rich snow and ice samples. Overall, the composite of parallel extractions, yielded up to 2 times more molecular formulas than those of the sequential extractions, suggesting that the OM extracted by HW, HCl, and NaOH combined were more diverse than the OM fraction obtained through the RTW-ACN and RTW-MeOH extraction. In all samples, the parallel extract composite list (Table S11) revealed relative abundances of 46–52% for unsaturated aliphatics and 41–44% for highly unsaturated compounds, while aromatics and polycyclic aromatics were lower in abundance (5–9% and <1.6%, respectively). Among the identified compounds, N-containing compounds were up to 3 times and 96 times higher in relative abundance than S- and P-containing compounds. Furthermore, the molecular formulas related to black carbon-derived polycyclic aromatics were up to 2.5 times higher in relative abundances in the RTW-ACN and RTW-MeOH extractions than the composite of the parallel extractions. However, it is worth noting that our sequential extractions selectively targeted only a smaller proportion (6–11%) of the total POM (Figure 2a,c), and the black carbon-derived polycyclic aromatics are found in high relative abundance within this limited pool.

Hence, the parallel extractions outperformed the sequential extractions in terms of overall OC yields and, when combined, also provided a broader coverage of chemical diversity. In addition, the inorganic solvents utilized in the parallel extractions primarily targeted acid–base-soluble organic compounds. In these extractions, OM loosely adsorbed onto mineral surfaces, OM associated with amorphous Si- Al- and Fe-oxides, as well as OM, stabilized by humic and clay-OM complexes, were likely targeted.^27,28,39,50,62^ Thus, this parallel extraction protocol allows for insights not only into the chemical properties of the POM pool but also into the nature of OM stabilization onto amorphous phases and mineral surfaces. Therefore, for our specific objectives of maximizing the recovery of OM bound to inorganic particulate surfaces and obtaining a comprehensive representation of the chemical diversity in these supraglacial systems using FTICR-MS, the parallel solvent extractions with HW, NaOH, and HCl are the most effective.

### POM Composition of Supraglacial Snow and Ice Samples

**Figure 4:**
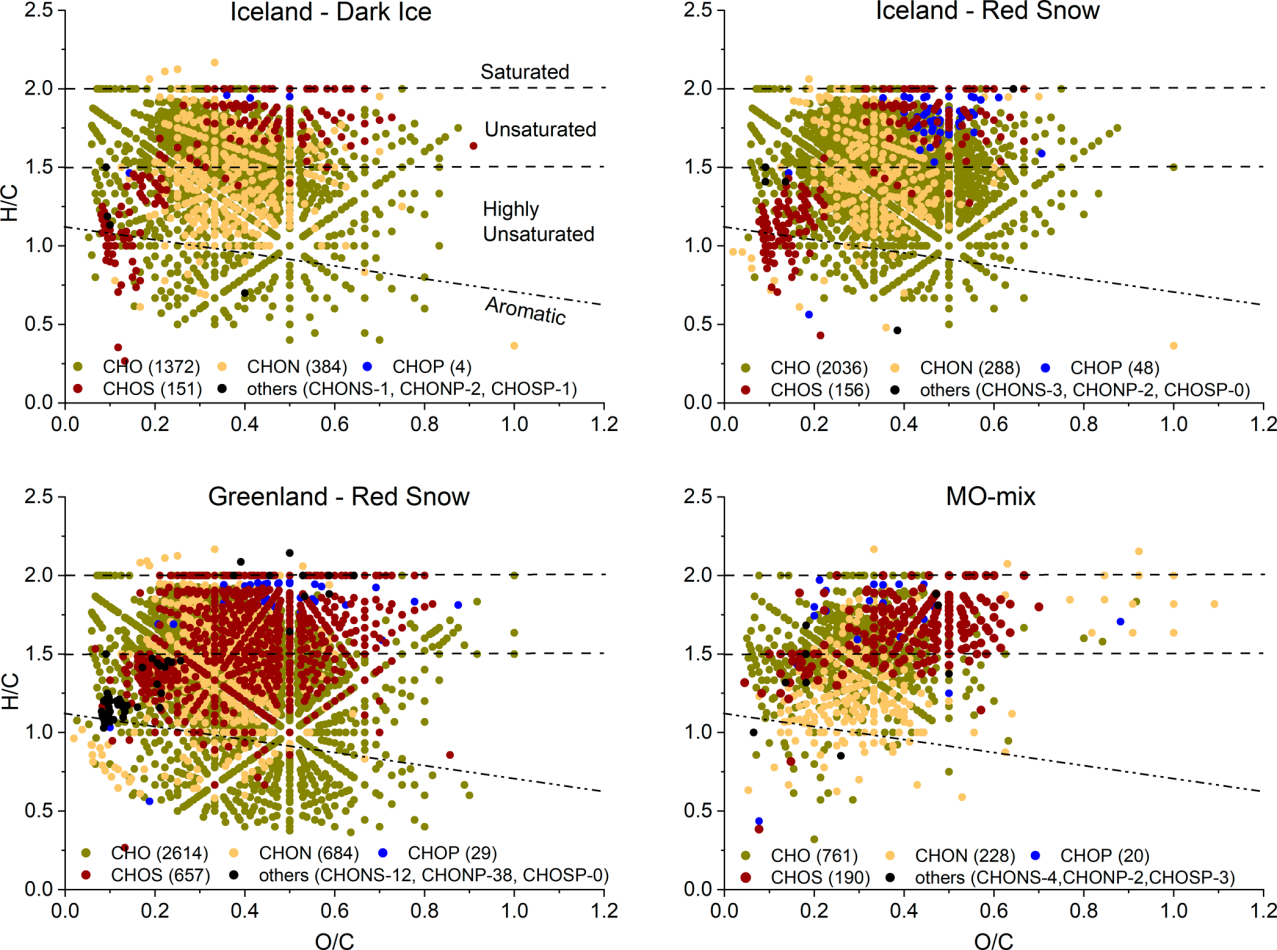
Molecular composition of POM isolated from Dark ice (Iceland), red snow samples (Iceland and Greenland), and an artificial reference mineral–organic mixture (MO-mix). This is a composite van Krevelen diagram generated by combining the unique peaks extracted by HW, NaOH, and HCl, as well as the peaks that overlapped between these solvents in the parallel extractions.

Comparison between the composite molecular compositions from the parallel extractions for the snow and ice samples from each location showed apparent differences between location and habitat type (Figure 4). The molecular composition of the red snow POM was characterized by a higher degree of unsaturation (DBE_w_), and *m*/*z*_w_ than the dark ice POM (Red snow: DBE_w_ 5.4, *m*/*z*_w_ 373; Dark ice: DBE_w_ 4.8; *m*/*z*_w_ 326). Despite the commonalities in bulk properties of the red snow samples from Iceland and Greenland, there were differences in the distribution of biochemical compound classes reflecting differences in microbial communities and mineralogical compositions in their originating environments. The sample from Greenland exhibited a higher number of molecular formulas and molecular diversity (Table S11). It featured a distinctive composition marked by a 2 to 19 times higher number of molecular formulas containing N, S, and P (CHON, CHOS, and CHONP; Figure 4) and a 10 times higher number of black carbon-derived polycyclic aromatics (Table S11). As POM composition is intricately linked to the microbial communities that dominate the biomass in a sample,^61,93,94^ differences in microbial communities inhabiting surface ice and snow habitats in Greenland and Iceland,^4,32,34^ may explain differences in POM composition across different habitats. For example, algal taxa constitute a larger proportion of the eukaryotic community in the red snow sample from Greenland (ca. 60%)^95^ compared to the samples from Iceland (under 20%),^32^ suggesting a relatively higher abundance of eukaryotic heterotrophs in the Iceland samples. N-, S-, and P- containing compounds have been previously linked with OM produced and/or reworked by microbial communities,^61,96,97^ with red snow- and glacier ice alga-rich habitats exhibiting distinct OM compositions.^61^ A high S content is also associated with microbial EPS,^98^ which in our samples binds the algae thriving on snow and ice surfaces to mineral-rich particulates. The EPS composition invariably varies between different microalgal species and even within strains of the same species.^99^ Thus, the diversity in microbial communities and their metabolic byproducts across different environments leads to distinct POM compositions. Additionally, variations in OM deposition from atmospheric sources, driven by differences in regional aerosol origins and transport pathways, may further contribute to the observed differences in OM composition between Greenland and Iceland.^100−102^ Principal component analysis based on the relative peak intensities of the composite formulas (Figure S7) shows a clear separation between the samples. The distinct clustering suggests that each sample possesses a unique molecular profile and that the variations in their organic composition contribute to the observed separation in the multivariate space. This variation of POM composition was likely driven not only by the composition of the microbial communities and associated EPS, but also by the variable mineralogical and geochemical signatures (e.g., particle size distribution, mineralogy, nutrient, and trace element supply, photosynthetic activity, OC inputs, etc.,) characteristic of each snow and ice environment. Although a full interpretation of these data is beyond the scope of this paper, our methodology has led to a first data set that helps better define sources of POM and OC cycling on glacier and ice sheet surfaces.

**Figure 5:**
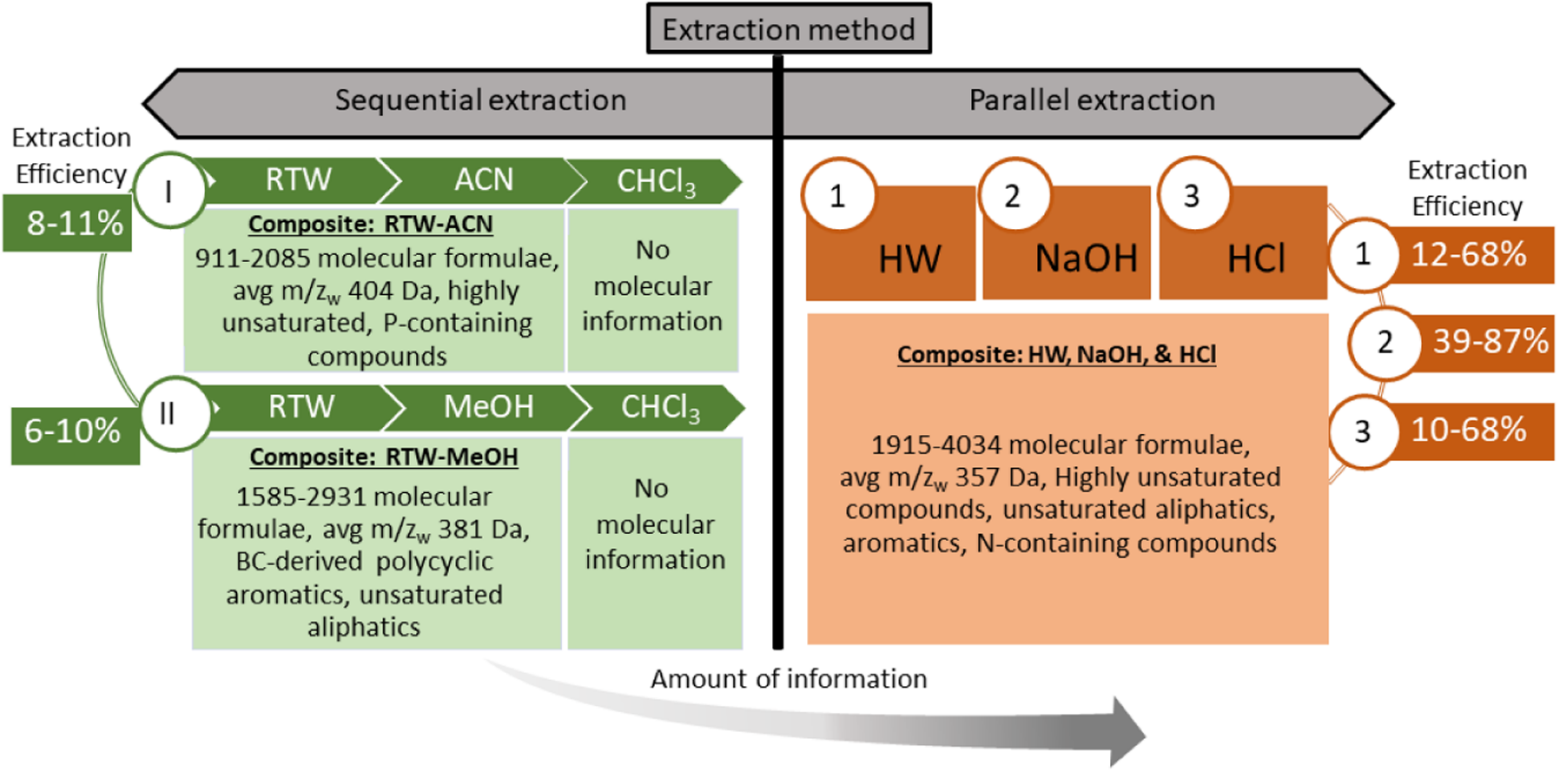
A schematic showing the amount of OC and the predominant glacial POM pool targeted by different extraction methods.

In summary, our highly effective and comprehensive protocol for extracting and characterizing OM within the mineral–organic matrix from snow and glacier ice surface samples involves parallel inorganic solvent (HW, HCl, and NaOH) and sequential organic solvent (RTW-ACN–CHCl_3_ and RTW-MeOH–CHCl_3_) extractions, followed by FTICR-MS analysis. We show that parallel extractions result in up to 8 times higher extraction efficiency than sequential extractions. Moreover, the combined molecular formulas in parallel extractions offer broad coverage of the chemical diversity, as each solvent extracts a complementary set of compounds, providing a highly representative description of the inherent diversity of particulate-bound OM (Figure 5) in alga-rich snow and bare-ice habitats. The snow and ice habitat type can also guide protocol selection if one is constrained by POM sample amount or FTICR-MS analysis costs. For example, HCl extractions yielded significantly fewer molecular formulas from red snow than from dark ice, so, parallel HW and NaOH extractions may be sufficient to cover the largest proportion of the POM chemical signatures for this habitat type. Our results also highlight how differences in the particulates’ mineralogical composition and associated differences in the strength of the organic-mineral interactions impact OM recovery. Nevertheless, we document how the molecular compositions of the extracted OM capture the chemical heterogeneity between samples and differentiate both based on location and habitat. These differences were mirrored in the distinct microbial community compositions and differences in OM inputs in Iceland and Greenland. Ultimately, we provide an essential first step in quantifying and understanding the molecular intricacies of the particulate-bound organic pool on glacier surfaces through a systematic and adaptable framework that can be used for other polar matrices, such as glacier flour, river sediments, or permafrost soils. Our protocols are easily transferrable because they deliver a clear path to characterize POM, thus offering valuable insights into its composition and influence on ecosystem dynamics in glacier environments, now and in a future warming climate scenario.

## Supporting Information Available

It includes a text file with details on sample collection and processing, cultivation and harvesting of algal biomass, preparation of the mineral–organic mix, extraction procedures, assessment of extraction method effectiveness, TOC analysis of particulates, DOC analysis of extracts, ESI-FTICR MS analysis, and data processing. Additionally, it includes figures on extraction efficiency and DOC_ex_ concentrations measured in the extracts, variations in the abundance of molecular categories identified in each solvent extract and sample, a scaled Venn diagram comparing the unique and common molecular formulas identified in the sodium hydroxide and sodium pyrophosphate extracts, principal component analysis biplot, and references as mentioned in the text (PDF)
Details of the samples used in this study, organic matter pools targeted by the different chemical solvents and chemistry data for each sample are provided in tables (XLSX)
