# Supplementary material for: Extraction Strategies for Profiling the Molecular Composition of Particulate Organic Matter on Glacier Surfaces
Source: Environ Sci Technol. 2025 Feb 27;59(9):4455–68. doi: 10.1021/acs.est.4c10088 (PMC11912203; doi:10.1021/acs.est.4c10088)
Supplement: Supplementary file 1 — es4c10088_si_001.pdf [file es4c10088_si_001.pdf]

Supporting Information for:

Extraction strategies for profiling the molecular composition of particulate organic matter on glacier surfaces

Runa Antony[1,2*], Pamela E. Rossel[1], Helen K. Feord[1], Thorsten Dittmar[3], Martyn Tranter[4], Alexandre Magno Anesio[4] and Liane G. Benning[1,5]

[1]GFZ Helmholtz Centre for Geosciences, D-14473 Potsdam, Germany

[2]National Centre for Polar and Ocean Research, Ministry of Earth Sciences, 403804 Goa, India

[3]University of Oldenburg, Institute for Chemistry and Biology of the Marine Environment, D-26046 Oldenburg, Germany

[4]Department of Environmental Science, Aarhus University, 4000 Roskilde, Denmark

[5]Department of Earth Sciences, Freie Universität Berlin, 12249 Berlin, Germany

*Corresponding author e-mail: rantony@gfz.de

The supporting information has 15 pages and includes 10 method texts, 7 figures, and references as mentioned in the text.

Supporting Information

Sample collection and processing

The snow and ice samples used in this study were from particulates collected in 2018 and 2019 from algae-dominated glacier surfaces in Iceland (Langjökull, Snæfellsjökull)[1,2] and Southeast Greenland (Mittivakkat, Heim, and Bruckner glacier).[3,4] Sample ID and location details are provided in Table S1. We characterized two types of samples, namely 'red snow' or 'dark ice,' depending on the prevalence of red snow algae or dark purple glacier ice algae. Both sample sets exhibited a total organic carbon (TOC) content of 0.3-6.4% C. In addition, we prepared an artificial mineral-organic reference material using mineral standards that mimic the mineral composition of Icelandic glaciers[5] and the Greenland ice sheet[6]. The organic component of the mineral-organic mixture included EPS-producing microalgae from a laboratory culture (see 'Mineral-organic mix' below for details) and reference graphitic carbon (Reference Material USGS24) as a proxy for black carbon to mimic the light-absorbing component of POM. We used this mineral-organic mix to validate our extraction methods by assessing whether the extracted OM represented the components of the artificial reference material, specifically the OM from algal biomass and black carbon. Black carbon comprises a continuum of organic compounds with varying reactivity, from lightly charred, labile molecules to highly condensed soot and refractory graphite.[7,8] We acknowledge that although graphitic carbon is a thermally altered byproduct of biomass burning, it lies at the far end of the combustion continuum, and differs in biological reactivity and solubility compared to soot, constraining our data interpretation. Full details of the preparation and composition of our artificial reference mineral-organic mixture are given below. Solvent extracts from the mineral-organic mix and three representative samples, GR19-MIT1 (Greenland red snow), IS19-13 (Iceland red snow), and IS19-14 (Iceland dark ice) with low particulate OC content (<1%) were selected to optimize our molecular characterization methods. We focused on these low OC samples to validate the robustness of our methods as supraglacial environments are highly oligotrophic and most often contain low OC content.[1,5,9,10] This allowed us to gain meaningful insights into the composition and dynamics of OM in these systems.

Samples were collected with conditioned metal trowels and transferred directly into ashed glass jars and, after melting at ambient air temperatures (usually within 2-4 h), filtered through 0.7 µm ashed GFF filters using a glass filtration unit. The particulates were transferred into ashed glass vials and frozen until returned to the home laboratory. There, they were freeze-dried until further use. The freeze-dried particulate samples were flash-frozen in liquid nitrogen for 10 min and ground using a Retsch MM2000 ball mill, which rotated a stainless-steel bowl with tungsten-carbide balls at 30 Hz for 1.5 min to ensure lysing of all cells and to obtain a fine powder.

Cultivation and harvesting of algal biomass

For the biomass component of the mineral-organic mixture, two algal strains, *Chlamydomonas reinhardtii* (SAG 33.89) and *Microglena*.cf. sp. -002b (CCCryo 002b-99), were cultivated. *C. reinhardtii*, a fast-growing EPS-producing alga,[11] was cultured in a Tris-Acetate-Phosphate medium at 20°C, while the cryophilic *Microglena* sp. from the Arctic, also EPS-

producing, was grown in a 3N BBM medium at 4°C. Cultures were grown in glass flasks under shaking at 120 rpm. Upon achieving optimal cell density, as ascertained through visual observation, cells were harvested by centrifugation at 1500 ×g for 5 min. The resultant cell pellets underwent two successive wash cycles, involving re-suspension in ultra-pure water followed by subsequent centrifugation. The final cell pellets were reconstituted in a minimal volume of ultra-pure water, transferred to a glass petri dish, and desiccated in an oven at 45°C until completely dry.

Mineral-organic mix

The microalgal biomass was gravimetrically mixed with a graphitic carbon reference material (USGS24) to obtain an organic mixture with a total carbon content of <1%, with the cellular material accounting for 95% of this total carbon mass and the rest from the graphitic carbon reference material. This was done assuming that (a) 100 mg of the biomass dry weight comprised 35 mg of carbon, a value set to be equivalent to the TOC content of algal and EPS-rich particulates collected previously from supraglacial high algal environments[1,3,12] and (b) that 96% of the mass of the black carbon-derived reference material is composed of elemental carbon (based on the USGS24 data sheet). Subsequently, a mineral mix was prepared from pure endmember materials that were commercially sourced amorphous SiO_2 (80%, CAS 65997-17-3), kaolinite clay (18%, CAS 1318-74-7), and hydroxyapatite (1%, CAS 12167-74-7) from Sigma-Aldrich and laboratory-synthesized goethite and hematite (1%) prepared following Cornell and Schwertmann.[13] These mineral phases were gravimetrically mixed to form a mineral mixture that was used to generate the final mineral-organic mixture.

The final mineral-organic mix was prepared in glassware that underwent baking at 450°C for 4 h, ensuring the elimination of potential OC contaminants. Low TOC, ultra-pure MilliQ water (18.2 MΩ) was added to a glass beaker containing precisely weighed organic and mineral constituents. This yielded a slurry that was agitated at 120 rpm for 15 h at room temperature to facilitate binding organic compounds to the minerals. The resulting mixture was dried in an oven at 105°C for 2.5 h, and large clumps were gently pulverized and homogenized directly within the beaker using an agate pestle. The TOC concentration of the milled mineral-organic mixture was determined to be 0.3 wt % C, based on an analysis using a Shimadzu high-sensitivity TOC-L_{CSH} analyzer coupled with a suspended solids measurement kit.

Extraction procedures

We tested two sequential extraction protocols that target different fractions of OM based on solvent polarity, each carried out in triplicate using 100 mg of freeze-dried particulates: (1) room temperature water(RTW)-acetonitrile(ACN)-chloroform($CHCl_3$) in sequence and (2) RTW-methanol(MeOH)-chloroform($CHCl_3$) in sequence. OM stabilized on mineral surfaces via multiple adsorption mechanisms, was targeted using four inorganic solvents with varying pH and selectivity in a parallel extraction method: hot water (HW, 80˚C; pH 6), hydrochloric acid (HCl, 0.5 M; pH 1), sodium pyrophosphate (NaPP, 0.1 M, pH 10), and sodium hydroxide (NaOH, 0.5 M; pH 13). Each solvent extraction, including method blanks (solvent without particulates), was performed in triplicate using 100 mg of milled particulates.

A previous study[14] demonstrated that extracts produced during sequential extraction using organic solvents similar to those employed in this study could be directly infused into the mass spectrometer without requiring solid-phase extraction (SPE) due to the lower levels of interfering salts in the organic solvent extracts. Furthermore, the SPE process inherently incurs some loss of OM.[15] In our study, the sequentially extracted samples had low OC concentrations, with only 6–11% of the total OC in the particulates being recovered. Avoiding SPE in this context minimized the loss of organic compounds without compromising the ability to acquire robust and high-quality Fourier transform ion cyclotron resonance mass spectrometry (FTICR-MS) data. In contrast, the fractions extracted in parallel extraction using inorganic solvents (i.e., HW, HCl, NaOH) contain high levels of salts and metals,[16,17,18,19] which can significantly interfere with FTICR-MS analysis. To mitigate these interferences, we subjected the parallel extraction samples to SPE to concentrate the OM and effectively remove salts and metals, ensuring compatibility with FTICR-MS analysis. The choice to use or bypass SPE was carefully determined based on the specific requirements of each extraction pipeline, balancing the need to maximize OM recovery and the quality of downstream molecular analyses. By doing this, our goal was not to compare SPE-treated samples with non-SPE-treated samples but rather to evaluate the efficiency and effectiveness of each extraction pipeline for recovering and characterizing OM from particulate-rich snow and ice samples.

Sequential extractions

All glassware used for the extraction was acid-cleaned (0.01 M HCl) and furnaced (450°C, 4h) before use. All reagents were LC/MS or trace metal grade. As our study focused on algae-dominated, carbon-poor, particulate-rich glacier snow and ice samples, we adapted and modified the sequential extraction protocols for low-carbon (<1%) sediments based on Tfaily et al.[14] utilizing room-temperature water and organic solvents. Water-extractable OM is among the most commonly studied fractions when analyzing the composition of OM in soils and sediments[14,20,21] and is frequently used as a proxy for *in situ* dissolved OC (DOC) in soil solutions.[22] Water extraction effectively isolates water-soluble organic compounds derived from microbial biomass, non-microbial organic sources loosely adsorbed on mineral surfaces, and OM bound within humic material.[23,24] In contrast, organic solvents are more effective at extracting hydrophobic domains within soil OM, and their use often reveals a greater abundance of low O/C ratio compounds in FTICR-MS analysis.[14,25] Additionally, the sequence in which solvents are applied during extraction significantly influences the chemical diversity of organic compounds detected. Tfaily et al.[14] demonstrated that sequential extractions beginning with water followed by organic solvents could increase the number of observed peaks by 2–4-fold, with each solvent selectively targeting distinct classes of compounds.

We tested two sequential extraction protocols that were carried out in triplicate using 100 mg of freeze-dried particulates: (1) room temperature water (RTW)-acetonitrile(ACN)-chloroform($CHCl_3$) in sequence and (2) RTW-methanol(MeOH)-$CHCl_3$ in sequence (Fig. 1a). The MeOH and $CHCl_3$ used in the second sequential extraction protocol was a 2:1 mixture of $CHCl_3$ and MeOH mixed with 0.2 its volume of water.[26] The upper MeOH layer and lower $CHCl_3$ layer in the biphasic system were collected separately, stored in glass bottles, and used for the water-MeOH-$CHCl_3$ sequential extraction. Method blanks comprising only the extracting solvent were processed in triplicates in a similar manner as the samples. The extracts were dried and redissolved in ultrapure water and methanol (1: 1 water/methanol (v/v)) before

analysis on the FTICR-MS, coupled with electrospray ionization (ESI) in negative ion mode. Direct injection into the mass spectrometer was preferred over purification via SPE, because of the low concentration of inorganic salts in the organic solvent extracts.[14] The extraction efficiency was estimated from the TOC concentration (wt %) of the particulates before extraction and the concentration of OC extracted by each solvent. Extracted DOC concentrations were determined on 81 extracts in triplicate by non-purgeable organic carbon analysis on a Shimadzu TOC-L_{CSH} analyzer calibrated using potassium hydrogen phthalate (Sigma-Aldrich) standards (see below for details). Particulate samples were analyzed in duplicate for TOC using an EA Isolink elemental analyzer coupled to a DELTA V Advantage Thermo isotope ratio mass spectrometer (see below for details).

Parallel extractions

The solvents for the parallel extractions were chosen based on their ability to selectively target distinct fractions of OM. Water, in particular, extracts a fraction that often exhibits the greatest diversity of chemical compound classes among OM fractions extracted from soils.[14] Additionally, the extraction temperature significantly influences the solubility and release of soil OM in water, with heating to 80°C markedly increasing the yield of OM compared to ambient temperatures.[18] This approach facilitates the investigation of a broad spectrum of organic compounds in particulate-rich samples. Acid hydrolysis, particularly with HCl, is commonly used to dissolve amorphous or reducible Fe-oxides[27] and release OM associated with these minerals through interactions such as co-precipitation and ligand exchange.[22] However, this approach only extracts OM soluble at acidic pH.[22] Alkaline extractions are effective at releasing OM from soils, resulting in high OC yield,[20] and are usually achieved through the use of 0.1 M NaOH[20,28,29] and NaPP.[20,16,19] Alkaline extractants deprotonate the acidic functional groups in OM, thereby enhancing its polarity and solubility in water,[19,30] releasing humic substances and OM that are strongly bound to active mineral surfaces and clay-OM complexes.[28] NaPP combines an alkaline pH with metal-chelating properties, enabling it to additionally solubilize OM stabilized by metal complexation.[16,17]

Assessment of extraction method effectiveness

The effectiveness of the extraction method was evaluated based on extraction efficiencies (measured by OC concentrations in the extracts and initial TOC content of the particulates) and the high compositional diversity, as indicated by the number of molecular formulae and the range of chemical classes detected using FTICR-MS analysis.

TOC analysis of particulates

Particulates from all natural samples were freeze-dried and then finely milled using a Retsch MM2000 ball mill with tungsten carbide balls. Samples were analyzed for total organic carbon using an EA Isolink elemental analyzer coupled to a DELTA V Advantage Thermo isotope ratio mass spectrometer. Reproducibility was better than 0.1 wt % C, based on repeated measurements of internal standards (Boden 3, HEKATECH).

DOC analysis of extracts

The amount of DOC extracted from the particulates (DOC_{ex}) in the natural samples and the mineral mixture using the different solvents was determined on a Shimadzu TOC-L_{CSH} analyzer calibrated using potassium hydrogen phthalate (Sigma-Aldrich) standards. To eliminate any carbon contribution from the organic solvents, the organic solvent extracts (100 µl) were dried down and then reconstituted in 10 ml of ultra-pure water acidified to pH 2. The water and inorganic solvent extracts (100 µl) were diluted in 10 ml of acidified ultra-pure water without drying down. Samples were vortexed to re-solubilize and mix the organic components. The pre-acidified samples were purged with CO_2-free air to remove inorganic carbon. The resulting sparged sample containing only non-purgeable organic carbon was injected in triplicates into a quartz combustion tube heated to 680°C where the OC is combusted to CO_2. A carrier gas then sweeps this CO_2 to the non-dispersive infrared gas analyzer (NDIR) detector, where the carbon dioxide content is measured. The detection limit is 7 µg L^{-1} and the standard error is less than 1.5% of the DOC concentration. Each triplicate extraction was analyzed at least in triplicate, and the results are presented as the average and standard deviation of the replicate measurements. For each extract, the value of the corresponding method blank comprising only the extracting solvent was subtracted from the DOC concentration before calculating the extraction efficiency (the proportion of the total amount of OC extracted). For calculation of the extraction efficiency, DOC_{ex} concentrations (mg C L^{-1}) were normalized to the weight of the particulates (mg), considering the amount of solvent and particulates that were used for the extractions. The extraction efficiency (ExE, in %) was calculated as the amount of OC (mg C) extracted from the particulates (C_{ext}) divided by the initial TOC content of the particulates before extraction (C_{in}) as follows:

$$ExE = \frac{C_{ext}}{C_{in}} \times 100$$

ESI-FTICR MS analysis and data processing

Molecular characterization was carried out using a Solarix 15T FTICR-MS (Bruker Daltonic) equipped with an Apollo II (Bruker) electrospray ionization source. We chose ESI as it is the most widely used ionization source for OM molecular characterization across diverse environmental samples. Specifically, we selected ESI in negative ion mode because prior studies examining OM in low OC (<1%) soils and sediments, using extraction solvents similar to ours, predominantly employed this mode[14,20,21,25] to obtain information on OM composition at the molecular level. Similarly, we opted for negative ion mode in our analysis to maintain consistency with previous studies and ensure comparability of results. Each duplicate extraction was analyzed on the FTICR MS in duplicate (technical replicates) in the negative ion mode. The diluted extracts were infused at 120 µL h^{-1}, and the ions were accumulated in the hexapole for 0.2 sec before transfer into the ICR cell. The mass spectra for each sample were accumulated with 200 scans. A total of 237 mass spectra were generated that were externally and internally calibrated using the arginine cluster and known molecular mass peaks in the sample over the entire mass range from 100-1000 m/z. Instrument performance was checked by repeatedly analyzing the Suwannee River Fulvic Acid (SRFA) reference standard between sample runs. The mass spectra of SRFA showed consistent profiles with no detectable variability over time, demonstrating the instrument's high

performance and stability during our analyses. After calibration, molecular formulae above the method detection limit (MDL) of 3 were assigned using ICBM-OCEAN, which removes noise peaks while retaining analyte peaks with low signal intensities, aligns masses across all spectra, and allows for a more precise formula assignment with the help of isotopologues.[31] Sample junction was conducted in fast join mode (0.5 ppm sample tolerance), with a recalibration tolerance of 0.5 ppm for formulae present in more than 1% of the samples. The minimum signal-to-MDL ratio used as the basis for recalibration was 5, utilizing the mean recalibration mode. Molecular formulae with isotope ratio mismatch above a signal-to-MDL ratio of 5 were excluded. Isotope tolerance was set at 1000 ‰. Molecular formulae were assigned with an error of < 0.5 ppm for the following combinations of elements: C_{0-100}, O_{0-50}, H_{0-200}, N_{0-4}, S_{0-2}, and P_{0-1}. Outliers were identified and removed using multiple approaches. In ICBM-OCEAN, an outlier test was applied to exclude peaks that deviate from the expected relationship between m/z and resolution. Additionally, resolving power vs. Kendrick mass plots and Kendrick mass defect vs. nominal Kendrick mass plots[32] were used to detect and eliminate peaks that fell outside predictable chemical patterns. Molecular formulae were further filtered using chemical plausibility rules, including the NSP rule, and other filtration criteria such as disallowing the combination of >3 N, S, or P atoms per molecule, and formulae with elemental ratio O/C=0 and O/C>1.1; and H/C>2 and N=0.[31,33] The homologous series network approach was applied to remove double assignments, considering CH_2, CO_2, H_2, H_2O, and O.[34] After applying the ICBM-OCEAN and other filtration criteria, only formulae consistently detected in >5% of the analyses across the dataset and present in all four replicate measurements of each sample were retained. Molecular formulae identified in blank samples that underwent identical processing as regular samples were excluded to ensure the exclusion of potential contaminants.

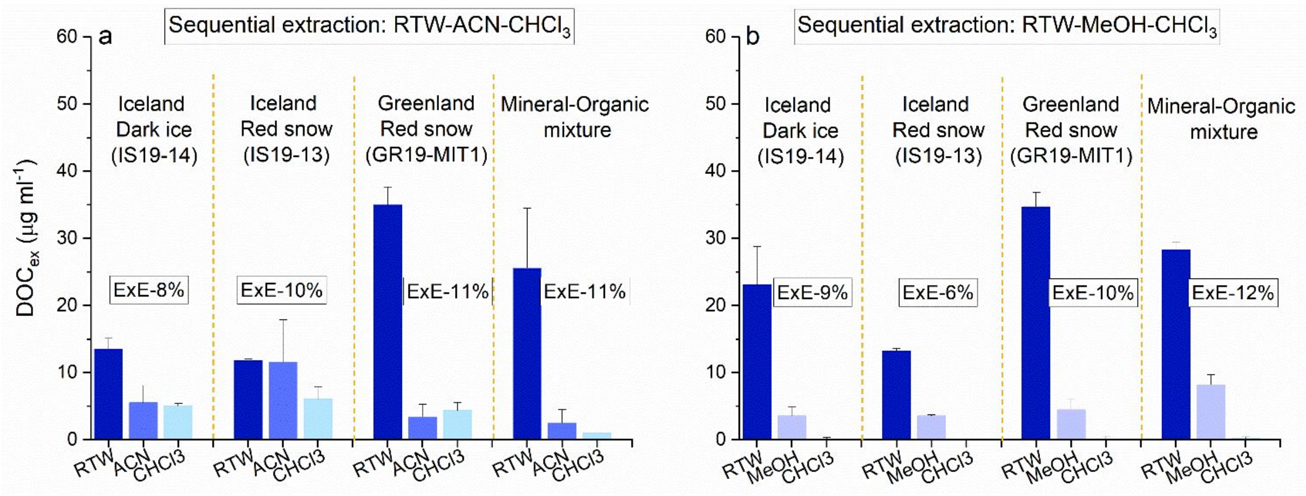

Figure S1. Dissolved organic carbon (DOC_{ex}) concentrations measured in the extracts following sequential extraction with a) RTW-ACN-$CHCl_3$ and b) RTW-MeOH-$CHCl_3$ used in sequence along with the corresponding extraction efficiencies (ExE). ACN, MeOH, and $CHCl_3$ refer to acetonitrile, methanol, and chloroform, respectively. Standard deviations represent triplicate extractions.

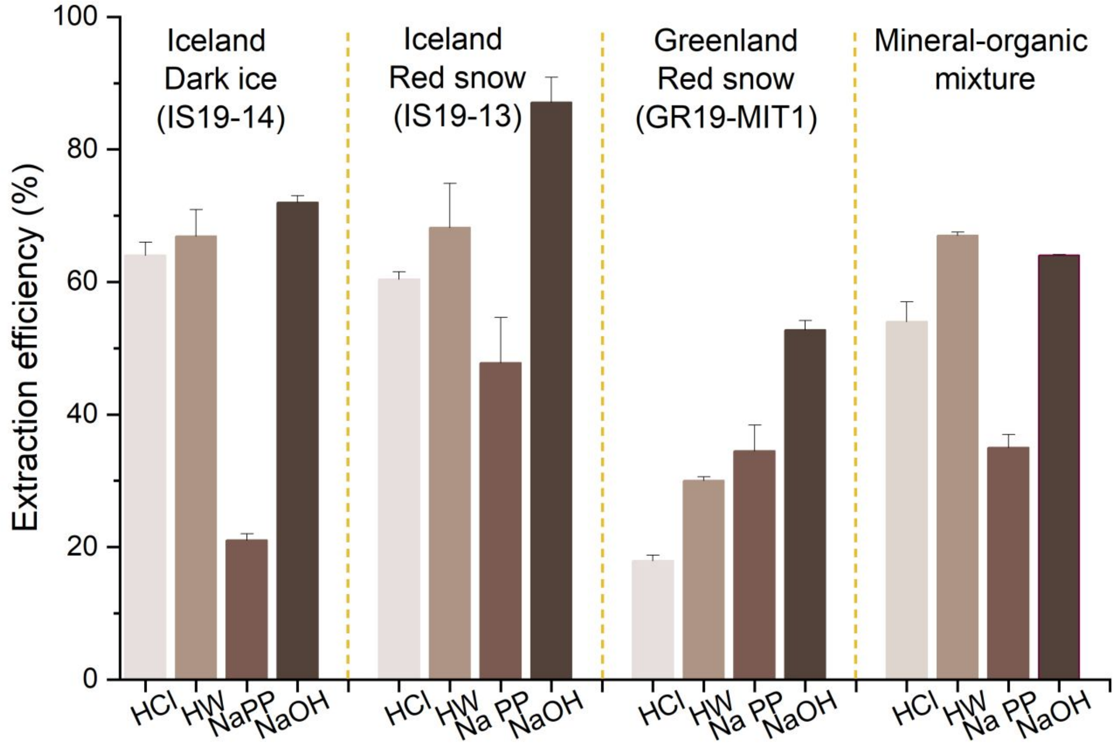

Figure S2. The average extraction efficiencies with parallel extractions using hydrochloric acid (HCl), hot water (HW), sodium hydroxide (NaOH), and sodium pyrophosphate (NaPP), for the mineral-organic mixture and three representative samples with low particulate carbon content (<1%). Standard deviations represent triplicate extractions.

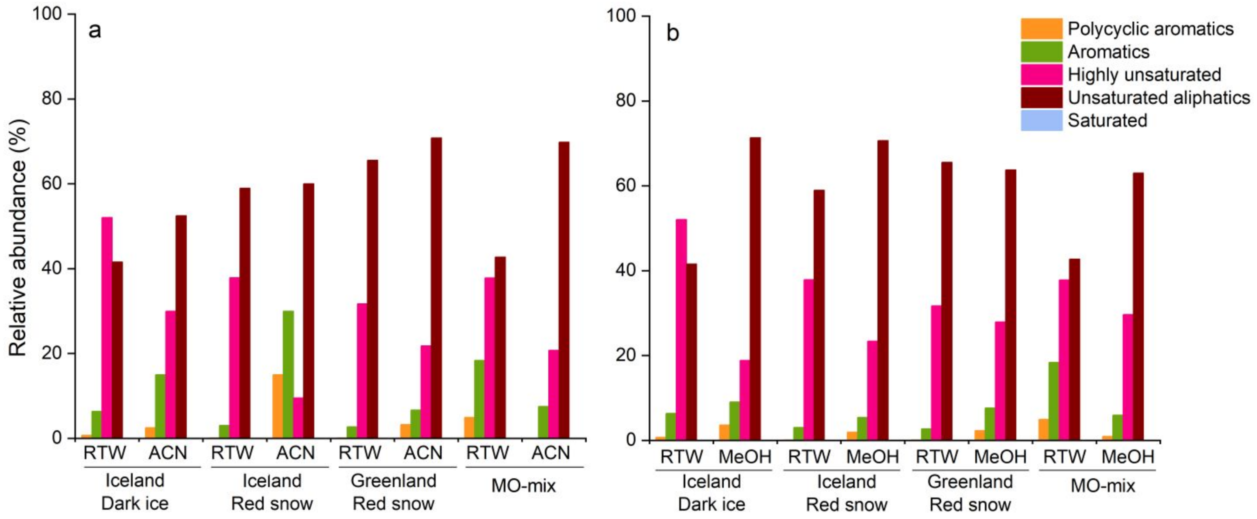

Figure S3. The contribution (in % abundance) of the different molecular categories relative to the total number of molecular formulae identified in each sample from Iceland (Dark ice: IS19-14; Red snow: IS19-13) and Greenland (GR19-MIT1) sequentially extracted with a) room temperature water (RTW) and ACN, and b) RTW and MeOH. MO-mix refers to the mineral-organic mixture. Polycyclic aromatics include aromatic compounds with $AI_{mod} \geq 0.67$ and $C \geq 15$, consistent with combustion-derived black carbon.[35] Refer to the methods section for details of compound classification.

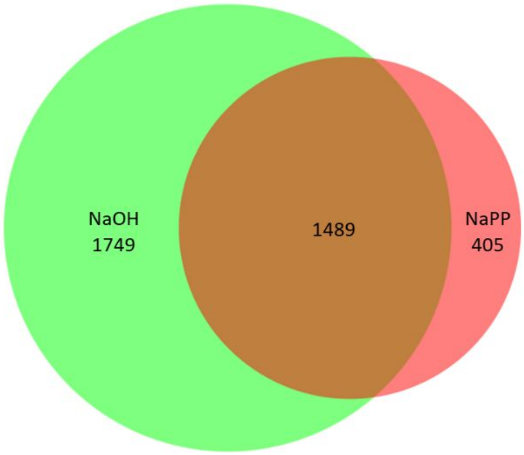

Figure S4. Scaled Venn diagram comparing the unique and common molecular formulae identified in the sodium hydroxide (NaOH) and sodium pyrophosphate (NaPP) extracts from the red snow sample from Greenland (GR19-MIT1).

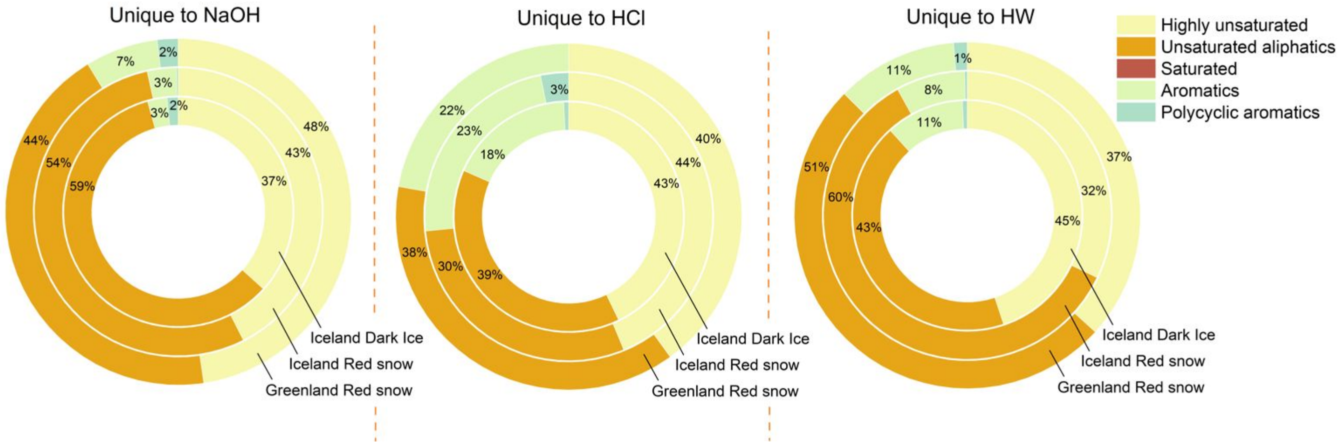

Figure S5. Variations in the abundance of compound classes (in %) relative to the total identified molecular formulae unique to each extract for the dark ice sample from Iceland (IS19-14) and red snow samples from Iceland (IS19-13) and Greenland (GR19-MIT1).

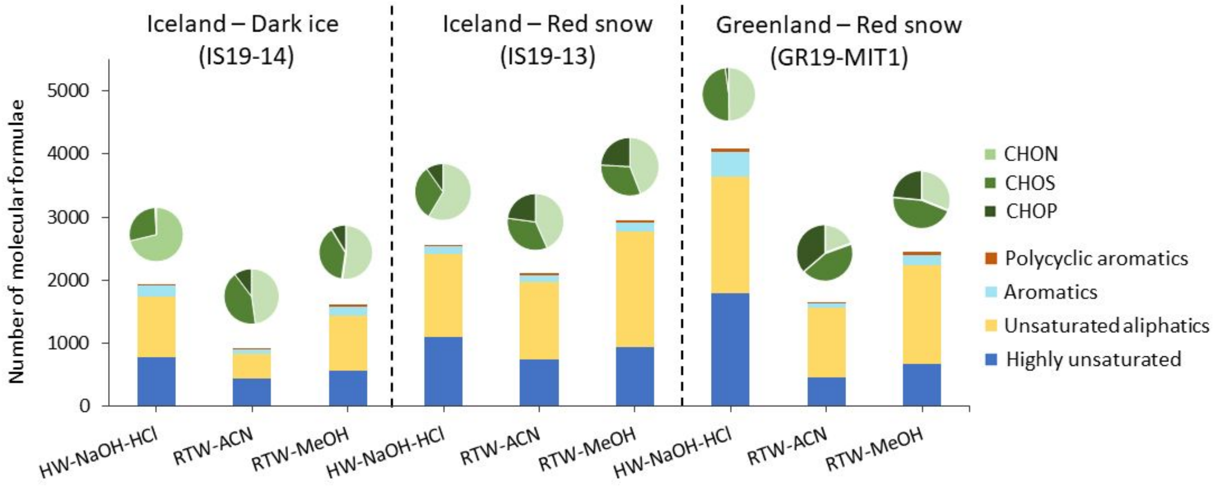

Figure S6. Variations in the number distribution of molecular categories identified in the composite of each of the three extraction protocols i.e., parallel extraction with hot water (HW), NaOH, and HCl; sequential extraction with room temperature water (RTW)-ACN; and sequential extraction with RTW-MeOH. The composite molecular list is generated by combining the unique molecular formulae extracted by each solvent in the protocol and the molecular formulae that are common between the solvents.

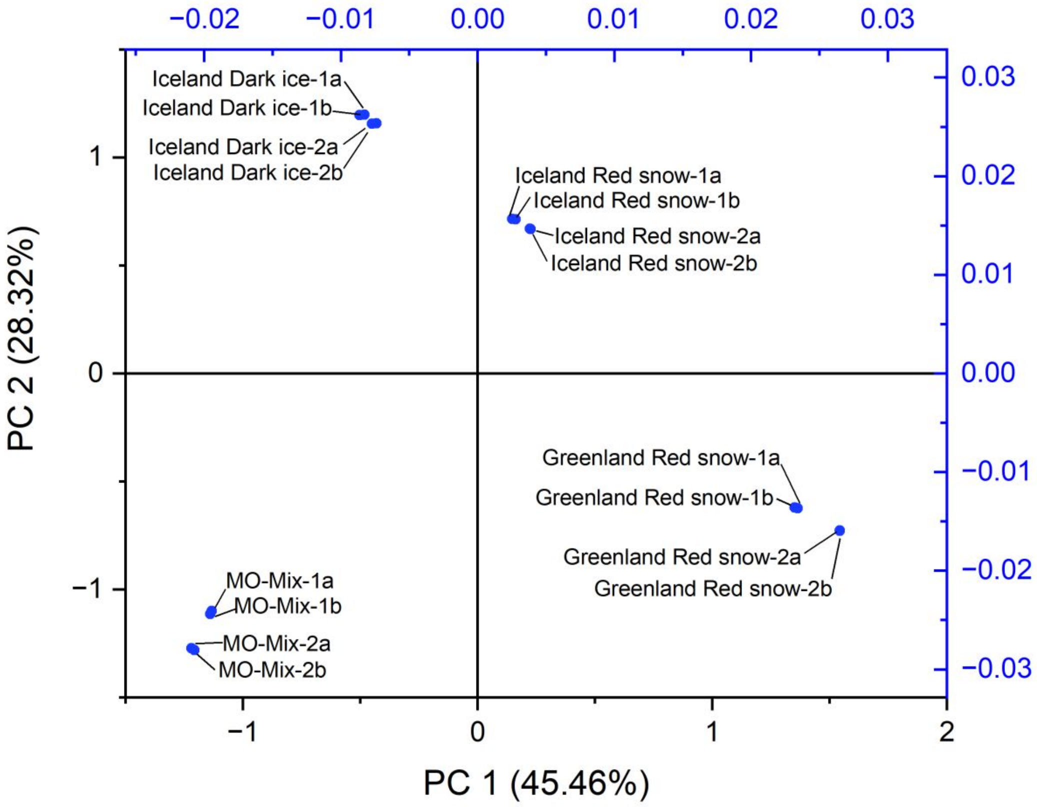

Figure S7. Principal component analysis biplot of the samples' scores. A data matrix was created using relative intensities of molecular formulae unique to HW, NaOH, and HCl and the common peaks between two or more solvents. Dark ice (IS19-14) and Red snow (IS19-13) from Iceland, Red snow from Greenland (GR19-MIT1), and the reference mineral-organic mixture (MO-mix) cluster into different areas, indicating statistically significant differences amongst sample types. Data are from replicate extractions (labeled 1, 2) and replicate FTICR-MS analysis of each extraction (labeled a,b).

References

1. Winkel, M.; Trivedi, C. B.; Mourot, R.; Bradley, J. A.; Vieth-Hillebrand, A.; Benning, L. G. Seasonality of glacial snow and ice microbial communities. *Front. Microbiol.* 2022, 13, 876848, DOI: 10.3389/fmicb.2022.876848.

2. Trivedi, C. B.; Keuschnig, C.; Larose, C.; Rissi, D.; Mourot, R.; Bradley, J. A.; Winkel, M.; Benning, L. G. DNA/RNA preservation in glacial snow and ice samples. *Front. Microbiol.*, 2022, 13, 894893, DOI: 10.3389/fmicb.2022.894893

3. Halbach, L.; Chevrollier, L. A.; Doting, E. L.; Cook, J. M.; Jensen, M. B.; Benning, L.G.; Bradley, J. A.; Hansen, M.; Lund-Hansen, L. C.; Markager, S.; Sorrell, B. K.; Tranter, M.; Trivedi, C. B.; Winkel, M.; Anesio, A. M. Pigment signatures of algal communities and their implications for glacier surface darkening. *Sci. Rep.* 2022, 12(1),17643, DOI: 10.1038/s41598-022-22271-4

4. Bradley, J. A.; Trivedi, C. B.; Winkel, M.; Mourot, R.; Lutz, S.; Larose, C.; Keuschnig, C.; Doting, E.; Halbach, L.; Zervas, A.; Anesio, A. M.; Benning, L. G. Active and Dormant Microorganisms on Glacier Surfaces. *Geobiology* 2023, 21, 244–261, DOI: 10.1111/gbi.12535

5. Lutz, S.; Anesio, A. M.; Edwards, A.; Benning, L. G. Microbial diversity on Icelandic glaciers and ice caps. *Front. Microbiol*. 2015, 6, 307.

6. McCutcheon, J.; Lutz, S.; Williamson, C. Cook, J. M.; Tedstone, A. J.; Vanderstraeten, A.; Wilson, S.; Stockdale, A.; Bonneville, S.; Anesio, A. M.; Yallop, M. L.; McQuaid, J. B.; Tranter, M.; Benning, L. G. Mineral phosphorus drives glacier algal blooms on the Greenland Ice Sheet. *Nat. commun.* 2021, *12*(1), 570, DOI: 10.1038/s41467-020-20627-w

7. Masiello, C. A. New directions in black carbon organic geochemistry. *Mar. Chem.* 2004, 92, 201–213

8. Coppola, A. I.; Wagner, S.; Lennartz, S. T.; Seidel, M.; Ward, N. D.; Dittmar, T.; Santín, C.; Jones, M. W. The black carbon cycle and its role in the Earth system. *Nat. Rev. Earth Environ.* 2022, *3*(8), 516–532, DOI: 10.1038/s43017-022-00316-6

9. Lutz, S.; Anesio, A. M.; Villar, S. E. J.; Benning, L. G. Variations of algal communities cause darkening of a Greenland glacier, *FEMS Microbiol. Ecol.* 2014, 89, 2, 402–414, DOI: 10.1111/1574-6941.12351

10. Stibal, M.; Šabacká, M.; Žárský, J. Biological processes on glacier and ice sheet surfaces. *Nat. Geosci.* 2012, 5, 771–774, DOI: 10.1038/ngeo1611

11. Li, C.; Zheng, C.; Fu, H.; Zhai, S.; Hu, F.; Naveed, S.; Zhang, C.; Ge, Y. Contrasting detoxification mechanisms of Chlamydomonas reinhardtii under Cd and Pb stress. *Chemosphere* 2021, 274, 129771, DOI: 10.1016/j.chemosphere.2021.129771

12. Lutz, S.; Anesio, A. M.; Edwards, A.; Benning, L. G. Linking microbial diversity and functionality of arctic glacial surface habitats. *Environ. Microbiol*. 2016, DOI: 10.1111/1462-2920.13494

13. Cornell, R. M.; Schwertmann, U. The Iron Oxides: Structure, Properties, Reactions, Occurrences, and Uses. WILEY-VCH Verlag GmbH & Co. KGaA, Weinheim, 2003, DOI: 10.1002/3527602097

14. Tfaily, M. M.; Chu, R. K.; Toyoda, J.; Tolić, N.; Robinson, E. W.; Paša-Tolić, L.; Hess, N. J. Sequential extraction protocol for organic matter from soils and sediments using high-resolution mass spectrometry. *Anal. Chim. Acta* 2017, 972, 54–61, DOI: 10.1016/j.aca.2017.03.031.

15. Dittmar, T.; Koch, B.; Hertkorn, N.; Kattner, G. A simple and efficient method for the solid-phase extraction of dissolved organic matter (SPE-DOM) from seawater. *Limnol. Oceanogr.−Methods*, 2008, 6, 230–235, DOI: 10.4319/lom.2008.6.230

16. McKeague, J. A. An evaluation of 0.1 M pyrophosphate and pyrophosphate–dithionite in comparison with oxalate as extractants of the accumulation products in Podzols and some other soils. *Can. J. Soil Sci.* 1967, 47, 95–99

17. Hall, G. E. M.; Pelchat, P. Comparison of Two Reagents, Sodium Pyrophosphate and Sodium Hydroxide, in the Extraction of Labile Metal Organic Complexes. Water Air and Soil Pollution, 1997, 99, 217-223, DOI: 10.1023/A:1018317407840.

18. Curtin, D.; Beare, M. H.; Chantigny, M. H.; Greenfield, L. G. Controls on the extractability of soil organic matter in water over the 20 to 80°C temperature range. *Soil Sci. Am. J.* 2011, 75(4), 1423-1430, DOI: 10.2136/sssaj2010.0401

19. Lopez-Sangil, L.; Rovira, P. Sequential Chemical Extractions of the Mineral-Associated Soil Organic Matter: An Integrated Approach for the Fractionation of Organo-Mineral Complexes. *Soil Biol. Biochem.* 2013, 62, 57–67, DOI: 10.1016/j.soilbio.2013.03.004

20. Fox, P. M.; Nico, P. S.; Tfaily, M. M.; Heckman, K.; Davis, J. A. Characterization of natural organic matter in low-carbon sediments: Extraction and analytical approaches. *Org. Geochem.* 114, 2017,12-22, DOI: 10.1016/j.orggeochem.2017.08.009

21. Schmidt, F.; Koch, B. P.; Witt, M.; Hinrichs, K. U. Extending the analytical window for water-soluble organic matter in sediments by aqueous Soxhlet extraction, *Geochim. Cosmochim. Acta* 2014, 141, 83–96, DOI: 10.1016/j.gca.2014.06.009.

22. Bahureksa, W.; Tfaily, M. M.; Boiteau, R. M., Young, R. B.; Logan, M. N.; McKenna, A. M.; Borch, T. Soil Organic Matter Characterization by Fourier Transform Ion Cyclotron Resonance Mass Spectrometry (FTICR MS): A Critical Review of Sample Preparation, Analysis, and Data Interpretation. *Environ. Sci. Technol.* 2021, 55, 9637−9656, DOI: 10.1021/acs.est.1c01135

23. Leinweber, P.; Schulten, H.R.; Körschens, M. Hot water extracted organic matter: chemical composition and temporal variations in a long-term field experiment. *Biol. Fert. Soils* 1995, 20, 17-23, DOI: 10.1007/BF00307836

24. Gregorich, E.G; Beare, M.H; Stoklas, U; St-Georges, P. Biodegradability of soluble organic matter in maize-cropped soils, Geoderma, 113, 2003, 237-252, DOI:10.1016/S0016-7061(02)00363-4.

25. Tfaily, M. M.; Chu, R. K.; Tolić, N.; Roscioli, K. M.; Anderton, C. R.; Paša-Tolić, L.; Robinson, E. W.; Hess, N. J. Advanced solvent based methods for molecular characterization of soil organic matter by high-resolution mass spectrometry. *Anal. Chem.* 2015, 87(10), 5206-15, DOI: 10.1021/acs.analchem.5b00116

26. Folch, J.; Lees, M.; Stanley, G. H. S. A simple method for the isolation and purification of total lipides from animal tissues. *J. Biol. Chem.* 1957, 226, 497–509

27. Chao, T., Zhou, L., 1983. Extraction techniques for dissolution of amorphous iron oxides from soils and sediments. Soil Science Society of America Journal 47,225–232.

28. Olk, D.C.; Bloom, P.R.; Perdue, E.M.; McKnight, D.M.; Chen, Y.; Farenhorst, A.; Senesi, N.; Chin, Y.-P.; Schmitt-Kopplin, P.; Hertkorn, N.; Harir, M. Environmental and Agricultural Relevance of Humic Fractions Extracted by Alkali from Soils and Natural *Waters. J. Environ. Qual.* 2019, 48, 217–232.

29. Stevenson, F.J. 1994. Humus chemistry: Genesis, composition, reactions. 2nd ed. John Wiley & Sons, New York.

30. Kleber, M.; Eusterhues, K.; Keiluweit, M.; Mikutta, C.; Mikutta, R.; Nico, P. S. Mineral-Organic Associations: Formation, Properties, and Relevance in Soil Environments; Advances in Agronomy, Vol. 130; Academic Press, 2015, DOI: 10.1016/bs.agron.2014.10.005

31. Merder, J.; Freund, J. A.; Feudel, U.; Hansen, C. T.; Hawkes, J. A.; Jacob, B.; Klaproth, K.; Niggemann, J.; Noriega-Ortega, B. E.; Osterholz, H.; Rossel, P. E.; Seidel, M.; Singer, G.; Stubbins, A.; Waska, H.; Dittmar, T. ICBM-OCEAN: Processing Ultrahigh-Resolution Mass Spectrometry Data of Complex Molecular Mixtures. *Anal. Chem.* 2020, 92(10), 6832–6838, DOI: 10.1021/acs.analchem.9b05659

32. Hughey, A. C.; Hendrickson, C. L.; Rodgers, R. P.; Marshall, A. G. Kendrick Mass Defect Spectrum: A Compact Visual Analysis for Ultrahigh-Resolution Broadband Mass Spectra. Anal. Chem. 2001, 73, 4676–4681

33. Rossel, P. E.; Vähätalo, A. V.; Witt, M.; Dittmar, T. Molecular composition of dissolved organic matter from a wetland plant (Juncus effusus) after photochemical and microbial decomposition (125 years): common features with deep-sea dissolved organic matter. *Org. Geochem.* 2013, 60, 62–71, DOI: 10.3389/fmars.2020.00428

34. Merder, J.; Freund, J. A.; Feudel, U.; Niggemann, J.; Singer, G.; Dittmar, T. Improved mass accuracy and isotope confirmation through alignment of ultrahigh-resolution mass spectra of complex natural mixtures. *Anal. Chem.* 2019, 92(3), 2558–2565, DOI: 10.1021/acs.analchem.9b04234

35. Seidel, M.; Beck, M.; Riedel, T.; Waska, H.; Suryaputra, I. G. N. A.; Schnetger, B.; Niggemann, J.; Simon, M.; Dittmar, T. Biogeochemistry of dissolved organic matter in an anoxic intertidal creek bank. *Geochim. et Cosmochim. Acta* 2014, 140, 418-434.
